# Effects of the *A*+ intervention on elementary-school teachers’ social and emotional competence and occupational health

**DOI:** 10.3389/fpsyg.2022.957249

**Published:** 2022-10-12

**Authors:** Sofia Oliveira, Magda Sofia Roberto, Ana Margarida Veiga-Simão, Alexandra Marques-Pinto

**Affiliations:** Centro de Investigação em Ciência Psicológica, Faculdade de Psicologia, Universidade de Lisboa, Lisboa, Portugal

**Keywords:** elementary-school teachers, implementation quality, intervention efficacy, occupational health, online intervention, professional development, social and emotional learning

## Abstract

Teaching is, to date, one of the most prone jobs to experiencing occupational stress and burnout. Owing to burnout’s negative personal, social, organizational and economic impacts, researchers, practitioners and education policy leaders are interested in developing practices and interventions aimed at preventing/reducing its prevalence. With teachers’ main professional demands to be of a social and emotional nature, interventions designed with a view to promote teachers’ social and emotional competence appears to be particularly promising, positively impacting teachers’ well-being and personal accomplishment and contributing to a decrease in their psychological distress, namely emotional exhaustion. However, theoretical and empirically grounded interventions with ecological validity and specifically targeting teachers are still scarce. Thus, to bridge the previously identified gaps, the present study aimed to evaluate the efficacy and the quality of the intervention’s implementation of the *A+*, an online social and emotional learning intervention for elementary-school teachers. A quasi-experimental study was conducted with a total of 81 participants (96.3% female, *M_Age_ =* 46.21, *SD_Age_* = 4.82, *n* = 42 assigned to the experimental group) from three different school contexts. School clusters were similar in size, organizational structure and socioeconomic level, and as regards previous attendance at social and emotional learning interventions; however, they differed with regards to perceived organizational climate. Data on the efficacy of the *A+* was collected across four waves using a set of self-report questionnaires that assessed proximal variables (i.e., social and emotional skills) and distal variables (e.g., well-being, burnout symptoms), and analyzed through Robust Linear Mixed-Effects Models. Coefficient omegas suggested adequate reliability of the measures. Additionally, two trained observers completed an observation grid to evaluate the quality of the *A+* implementation (e.g., participant responsiveness, fidelity), with excellent inter-rater reliability. Results suggested that, over time, the *A+* had positive impacts across proximal (e.g., increased self-regulation, positive relationship, conflict management skills) and distal variables (e.g., increased emotional well-being, decreased occupational stress and emotional exhaustion symptoms) favoring the experimental group. However, results differed across the school contexts. These findings were accompanied by good implementation quality indicators, namely high fidelity in the delivery of the *A+* contents and high participants’ responsiveness. Despite its limitations, this study contributes to a growing body of research which reinforces the importance of investing in social and emotional learning interventions to prevent teachers’ burnout and improve their occupational health. Furthermore, it highlights the importance of implementation quality research as a component of program planning with a view to enhancing programs’ efficacy, as well as the need to adapt and consider context variables in research and practice.

## Introduction

Decades of research depict teaching as a highly demanding job which endorses the experience of chronic stress and burnout episodes (Maslach et al., 1996; Schaufeli and Buunk, 2003). Particularly, teachers are expected to cope with the daily challenges of their job (e.g., workload and time pressure, managing relationships with peers, school leaders, and students and their parents, dealing with criticism, classroom management) along with the new responsibilities that arise due to social changes (e.g., new teaching methods, curriculum content; Kyriacou, 2011). The period we have gone through in the last few years is a tangible example of this. Due to the SARS-CoV-2 pandemic outbreak, teaching demands have worsened in the last years, with teachers facing the need to adapt to new challenges, reinventing teaching methodologies and developing and perfecting their pedagogical, social, and emotional skills, whilst navigating adversity in their personal lives (e.g., Sokal et al., 2020; Kraft et al., 2021; Trinidad, 2021). Particularly, in Portugal, elementary-school teachers appear to have perceived the most teaching difficulties and decrease in their well-being in comparison with pre-school, middle and high-school teachers (Alves et al., 2021). Against this background, teaching is, to date, one of the professions with the highest risk of ill-health (European Commission, 2021), with many teachers presenting burnout symptoms (Varela et al., 2018; Marken and Agrawal, 2022). Consequently, teachers’ occupational health, well-being, and performance are negatively impacted (Maslach and Leiter, 2016). Moreover, due to the co-regulative nature of classrooms, teacher burnout also indirectly compromises students’ well-being and academic achievement (Jennings and Greenberg, 2009; Durlak et al., 2015; Gotlieb et al., 2022). In this scenario, to develop practices and interventions aimed at promoting teachers’ occupational health and well-being gains especial relevance (Granziera et al., 2021; Gotlieb et al., 2022).

Interventions seeking to reduce teachers’ burnout through the development of teachers’ resources have increased in the last decades. These interventions have been mostly individual-directed and adopted mainly cognitive-behavioral or, as observed more recently, mindfulness-based stress reduction strategies (Maricuţoiu et al., 2016). However, with regard to these interventions’ efficacy, findings are remarkably inconsistent and often narrowed down to reducing emotional exhaustion symptoms (Maricuţoiu et al., 2016). Therefore, literature has stressed the need to investigate new intervention approaches that would complement traditional stress-reduction interventions (Maricuţoiu et al., 2016; Iancu et al., 2018). In this respect, Social and Emotional Learning (SEL) interventions have shown promising results in promoting teachers’ occupational health and well-being (Oliveira et al., 2021a,b). Thus, with a view to contributing to the knowledge in this field, this study primary aims to evaluate the efficacy of the *A+*, an online SEL intervention program for elementary-school teachers. Moreover, following the literature in the field of intervention programs’ development and evaluation (Fernández-Ballesteros, 1996; Durlak et al., 2015), this study also aimed to explore the role of organizational climate and quality of intervention’s implementation (specifically the participants’ responsiveness impact) in the program outcomes.

### Impacts of social and emotional leaning interventions on teachers’ occupational health

Following the Job Demands and Resources (JD-R) model (Demerouti et al., 2001; Prieto et al., 2008), teachers’ occupational ill-health stems from a perception of excessive job demands (e.g., time pressure and workload, interpersonal conflicts, coping with change) accompanied by the absence of personal and job resources to face these job strains (e.g., lack of autonomy, lack of emotion and behavior regulation skills, lack of organizational and social support; Kyriacou, 2001, 2011; Schaufeli and Taris, 2014). Specifically for teachers, the main sources of teachers’ occupational stress and burnout are variables of a social and emotional nature (Kyriacou, 2001, 2011). Thus, teachers’ social and emotional competence (SEC; i.e., self-awareness, self-regulation, social awareness, relationship skills, and responsible decision-making; Durlak et al., 2015) have been highlighted as important protective factors to buffer against burnout and increase teachers’ occupational health (Jennings and Greenberg, 2009; Schonert-Reichl, 2017). Hence, in the last 15 years, a rapid increase of interventions aiming to develop teachers’ SEC, i.e., SEL interventions, has been witnessed (Oliveira et al., 2021a).

SEL for teachers is grounded in three main theoretical frameworks of reference (Jennings and Greenberg, 2009). First, the *Emotional intelligence theory* (Salovey and Mayer, 1990) which frames the five-core and interrelated domains of SEC that should be explicitly address within these interventions’ content. Second, the *Transactional model of stress and coping* (Lazarus and Folkman, 1984) which provides information regarding the main teacher-specific stressors and informs on coping strategies to include in teachers’ training. Lastly, the *Self-determination theory* (Deci and Ryan, 1985) which offers guidance on how to promote teachers’ motivation for behavior change and learning. Overall, SEL interventions aim to enhance teachers’ intra-and inter-personal development and their responsible decision-making skills, in the face of which they adapt and effectively respond to personal and professional challenges (Elias et al., 1997; Durlak et al., 2015). Empirical evidence has supported these interventions’ efficacy in promoting teachers’ SEC (e.g., emotional and behavioral regulation; e.g., Jennings et al., 2013, 2017, 2019; Carvalho et al., 2021). Moreover, prior research has also sustained these interventions’ indirect effects on reducing teachers’ psychological and physical discomfort, enhancing teachers’ personal and professional well-being, and work performance. Specifically, effects have been found in reducing teachers’ occupational stress and burnout symptoms (e.g., Roeser et al., 2013), ache-related symptoms, blood pressure and cortisol levels (e.g., Harris et al., 2016; Jennings et al., 2019), along with an increase of teachers’ self-care practices and sleep quality (e.g., Harris et al., 2016; Crain et al., 2017), job and life satisfaction (e.g., Crain et al., 2017) and well-being (e.g., Jennings et al., 2013, 2019; Carvalho et al., 2021). Furthermore, SEL interventions for teachers have been linked to an increase in teachers’ ability to manage their classrooms, providing greater emotional and instructional support for their students, and improving teacher-student interactions (e.g., Jennings et al., 2017; Carvalho et al., 2021). Taken together, these findings sustain that teachers who are more socially and emotionally competent are more effective in adapting and responding to personal and professional demands, displaying higher levels of occupational health and well-being, performance and positive interpersonal relationships. Recent meta-analyses have also sustained the promising contributions of these interventions in promoting teachers’ perceived SEC, mitigating their psychological distress (namely emotional exhaustion symptoms), and improving teachers’ well-being and personal accomplishment (Oliveira et al., 2021a,b). Additionally, due to the co-regulative nature of classroom interactions, socially and emotionally competent teachers also appear to foster their students’ SEC, well-being, and academic achievement (Jennings and Greenberg, 2009; Carvalho et al., 2021).

Overall, SEL interventions for teachers appear to support the development of teachers’ resources (namely personal and non-work specific), which are particularly important in mitigating the impact of teachers’ job demands (Bianchi et al., 2021). Particularly, as regards teacher burnout prevention, SEL interventions appear to be especially effective in promoting teachers’ personal accomplishment, when targeting (pre-)kindergarten and elementary-school teachers (Oliveira et al., 2021b). In a scenario resulting from the SARS-CoV-2 pandemic outbreak, where teachers’ social and emotional job demands (e.g., work-life balance, time management, workload, interpersonal relationships/conflict, emotional regulation; Sokal et al., 2020) were exacerbated to an unprecedented extent, SEL interventions for teachers are even more necessary (Gotlieb et al., 2022), particularly for elementary-school teachers (Alves et al., 2021).

Nevertheless, despite these promising contributions, prior literature signals the need to further invest in the development of theoretically and empirically grounded (Durlak et al., 2015) and culturally adapted interventions (Granziera et al., 2021), which has not yet been seen across the majority of SEL interventions for teachers (Oliveira et al., 2021a). Thus, in the context of this study, we aimed to evaluate the efficacy of the *A+*, an online SEL intervention program for elementary-school teachers. This study is part of a larger investigation trial aiming at the planning and evaluation of a culturally adapted, theoretically and empirically grounded SEL intervention program, specifically developed for Portuguese elementary-school teachers. In a previous stage, a needs assessment study ensuring empirical support, cultural adequation and ecological validity to the intervention’s contents and methodologies, along with a pilot study for the assessment of a trial version of the *A+*‘s social validity and efficacy was conducted (*see* Oliveira et al., under revision). In this stage, resorting to the *A+*’s expected efficacy and in accordance with prior literature findings, the following research hypotheses were established:

*H1:* The *A+* intervention program will enhance teachers perceived social and emotional competencies across time and when compared with the control group.

*H2:* The *A*+ intervention program will positively impact teachers’ self-care practices, sleep quality, and well-being (H2a) and negatively impact teachers’ occupational stress and burnout symptoms (H2b) across time and when compared with the control group.

Furthermore, in line with what has been seen in other intervention approaches with teachers (Iancu et al., 2018), SEL interventions for teachers have been mostly individual-directed not explicitly considering contextual-level factors (Oliveira et al., 2021a). Moreover, prior literature also stresses the need to develop more methodological robust studies aiming at SEL program evaluation (Oliveira et al., 2021a). Specifically, there is need to investigate time stability (i.e., maintenance, or not, of the intervention’s effects over time) and the possible sleeper effects (i.e., long-term lagged effects which require some incubation time and, so, are not immediately present at posttest) of SEL interventions, namely through follow-up assessments (Oliveira et al., 2021a). Additionally, it is important to consider the role of implementation quality variables (such as fidelity, quality, and participants’ responsiveness), since both have been less studied and may interfere with the interventions’ efficacy outcomes (Berkel et al., 2011, 2018; Humphrey et al., 2018; Oliveira et al., 2021a).

### The relationship between organizational climate and teachers’ SEC

The role of social and contextual dimensions is depicted consistently throughout the different theoretical frameworks which frame teachers’ SEL and occupational health. However, although different models (e.g., Collie’s (2020) social and emotional competence school model; Jennings and Greenberg’s (2009) prosocial classroom model; and Marchand et al.’s (2015) multilevel determinants of workers’ mental health model) describe the influence of contextual dimensions on teachers’ SEC, occupational health and well-being, research and practice which contemplates the impact of contextual variables is still scarce (Oliveira et al., 2021a). Thus, to date, literature continues to highlight the need to assess the role of contextual variables in teachers’ personal and professional outcomes (Schaufeli and Taris, 2014; Collie, 2017).

In the context of teachers’ occupational health and well-being, organizational climate has been a primary focus of research (Schaufeli and Taris, 2014). Organizational climate refers to the set of single characteristics that are perceived by the personnel, making each working context unique and influencing the workers’ behaviors (Hoy and Tarter, 1992). Due to its strong links to teachers’ emotions and behaviors (Collie, 2017), prior research has pointed to organizational climate as a predictor of teachers’ job satisfaction, efficacy, stress and burnout symptoms (Collie et al., 2012, 2018; Skaalvik and Skaalvik, 2018; Ford et al., 2019).

The work context characteristics have not only been directly linked to teachers’ occupational (ill-)health symptoms, but research has also sustained that subjective evaluation of the contextual variables determines the efficacy of different resources in responding to job demands (Taris et al., 2017). Recent studies also suggested a positive association between a closed or unhealthy organizational climate and lower personal resources (e.g., lower SEC) (Collie et al., 2012; Collie, 2017, 2020). Moreover, the demands that arose from the SARS-CoV-2 pandemic outbreak also impacted the contextual dimensions (namely organizational and social support) which were previously linked to teachers’ personal resources (e.g., SEC) and their occupational health (Sokal et al., 2020, 2021; Kraft et al., 2021; Trinidad, 2021). Thus, in the face of heightened job demands and teachers’ consequent increased vulnerability to occupational stress and burnout, interventions should consider contextual variables in their assessment needs, content development and efficacy evaluation (Schaufeli and Bakker, 2004).

Therefore, in this study, teachers perceived organizational climate was considered as one of the central variables of this study. Not only context-specific needs were accounted for during the development of the intervention’s contents and methodologies (*vide* section “Intervention” for further detail), as the impact of the organizational climate on teachers’ SEC was also assessed in this study. Therefore, the following research question was established:

*Q1: Does organizational climate predict the degree of teachers’ SEC pre-intervention?*

### The role of implementation quality in program evaluation

Since they may interfere with the intervention’s efficacy, prior literature on SEL for teachers signals the scarcity in assessing implementation quality variables as one of the main weaknesses of current investigation on the existing SEL programs (Berkel et al., 2011, 2018; Humphrey et al., 2018). Following the model proposed by Berkel et al. (2011, 2018), two core parameters are likely to interfere with the intervention’s outcomes: the facilitator behaviors (i.e., fidelity, quality, and adaptation) and the participants’ behaviors (i.e., responsiveness). To date, even scarce, prior studies on SEL for teachers have mainly addressed the impact of fidelity on program outcomes (Oliveira et al., 2021a). However, even when fidelity is ensured, participants’ responsiveness (i.e., their active participation/engagement and attendance) may directly impact participants’ individual learning and development (Humphrey et al., 2018; Schussler et al., 2020). Therefore, this study aimed to assess the *A+*‘s quality of implementation and its relationship with the intervention’s outcomes. Hence, the following research question was established:

*Q2: Was the A*+ *implemented with quality? If so, how does participants’ responsiveness impact the degree of teachers’ SEC post-intervention?*

**Figure 1:**
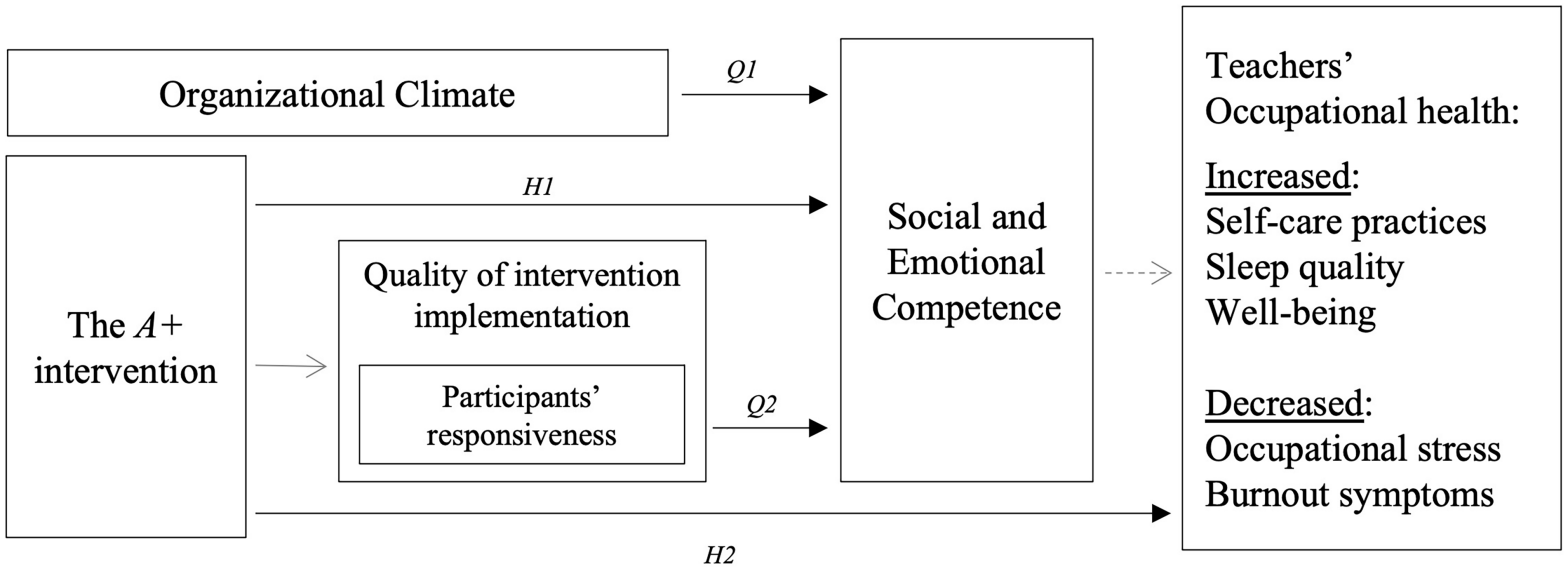
The proposed conceptual model of relationships between study variables. Due to sample size limitations, despite theoretically sustained, mediation analysis was not tested in this study.

Figure 1 depicts the proposed conceptual model of relationships between the variables under study.

## Materials and methods

### Participants

**Figure 2:**
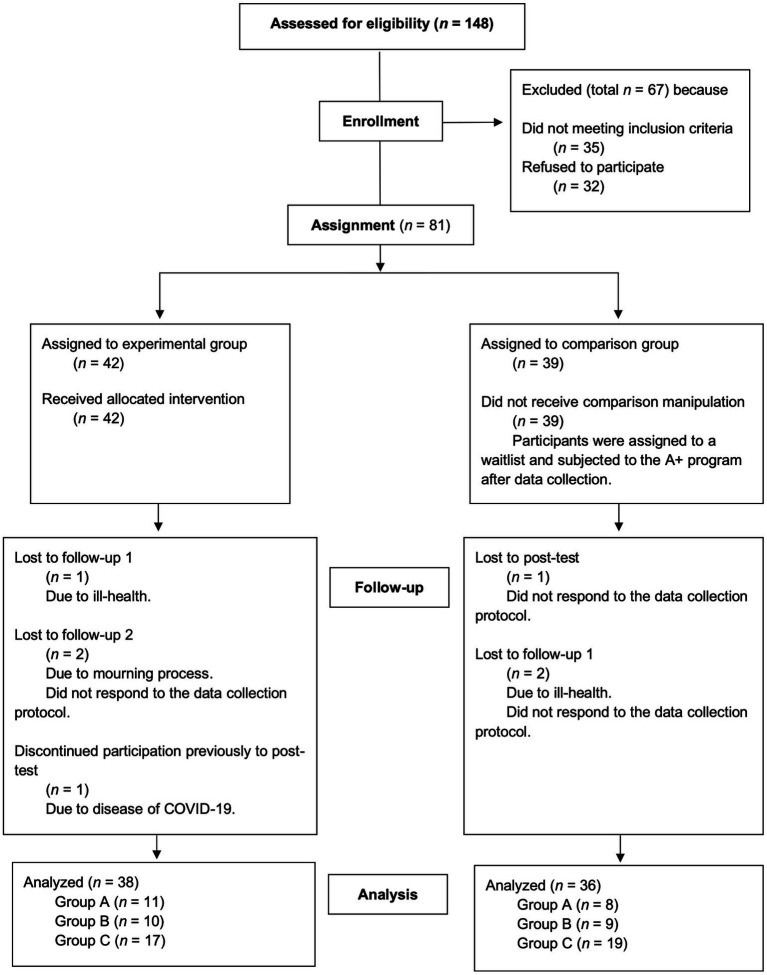
CONSORT flowchart.

Eighty-one elementary-school teachers (96.3% female, *M_Age_* = 46.21 years, *SD_Age_* = 4.82) enrolled in the study. The participants had a mean of 11.08 (*SD* = 8.18) years of teaching experience and were practicing in state elementary schools from three different school clusters (referred to as Clusters A, B and C) across the Lisbon Metropolitan Area. Table 1 depicts a detailed characterization of the participants between the school clusters. Prior to this study, 71.6% of the participants had never attended a SEL intervention. The three school clusters were similar in size and also in terms of organizational structure and socioeconomic level. The experimental group (EG) included 42 participants, whilst the waitlist control group (CG) was comprised of 39 participants. Thirty-eight teachers from the EG and 36 teachers from the CG completed all four data collection waves. The overall attrition rate (8.64%) and the differential attrition rate (1.81%) at follow-up 2 were low under the optimistic threshold (CONSORT flowchart is depicted in Figure 2).

**Table 1:** Participants’ sociodemographic characteristics between school clusters (percentage of the most frequent category, mean and standard deviation).

	Cluster A		Cluster B		Cluster C	
	%	*M* (SD)	%	*M* (SD)	%	*M* (SD)
Gender (Female)	100.00		100.00		92.30	
Age		44.60 (4.33)		47.36 (4.78)		46.38 (4.97)
Years of teaching experience in the school cluster		7.60 (6.53)		15.64 (8.07)		10.31 (8.04)
Highest Educational Qualification (Master)	80.00		59.10		69.20	
Frequency of prior SEL interventions (No)	70.00		72.70		71.80	

Despite the similarities on geographic location, size, organizational structure, and socioeconomic level, the three school clusters differed regarding perceived SEL needs and professional demands and resources. Professional demands and resources were acknowledged both on a personal level (i.e., individual characteristics which either hinder or promote teachers’ performance) and on a contextual level (i.e., aspects from teachers’ immediate work environment or indirect/cultural environment perceived to either hinder or promote teachers’ performance). In keeping with the findings of the prior study on the needs assessment of these contexts (Oliveira et al., under revision), we were able to understand that teachers from the Cluster A were those who identified the most professional demands at an indirect/cultural environment level (e.g., media, laws, social beliefs). At the same time, this was the school cluster where teachers perceived to have more personal resources (e.g., professional self-efficacy, work-life balance). Nevertheless, emotional regulation and, consequently, the experience of negative emotions and related symptoms, emerged has the primary risk factor of a personal level within this school cluster.

On the other hand, teachers from the Cluster B were the ones who described a greater balance between professional demands and resources. This was the school cluster to acknowledge the most protective work environment, with teachers identifying strong and supportive networks with their peers and the school leaders, and fewer risk factors in comparison with the Clusters A and C. Nevertheless, this was also, in comparison, the cluster where teachers made less references to personal resources, identifying emotional regulation as their greatest challenge.

Lastly, teachers from the Cluster C were those who recognized more professional demands, particularly at the personal (e.g., strains managing pedagogical relationships, emotional regulation, professional demotivation and turnover intention) and work environment (e.g., segregation of schools within the school cluster) levels. Complementarily, teachers from this school cluster referred to social support networks with peers as the strongest resource at the school level, although they also perceive a lack of contact with their peers. Full detail on the three clusters’ SEL needs, and perceived professional demands and resources are described in a previous qualitative study covering the needs assessment which underlie the *A+* development (*see* Oliveira et al., under revision).

### Measures

#### Evaluation of the intervention’s efficacy

The *Social and Emotional Competence Assessment Battery for Adults* (Oliveira et al., in press) was used to evaluate teachers’ SEC. This battery integrates three independent questionnaires in a total of 37 items. The *Intrapersonal competence questionnaire* consists of two scales: Self-awareness (7 items, e.g., “In my daily life, I am able to identify and name my emotions when they occur.”; 0.79 < *ω*_T1–T4_ < 0.82) and Self-regulation (8 items, e.g., “I can adapt (e.g., thinking differently) towards new information or situations.”; 0.80 < *ω*_T1–T4_ < 0.87). The *Interpersonal competence questionnaire* is composed of two scales: Positive relationship (8 items, e.g., “I give appropriate feedback (e.g., timely, constructive).”; 0.78 < *ω*_T1–T4_ < 0.83) and Conflict management (8 items, e.g., “In the face of a conflict with someone I know, I am able to listen carefully to what that person is saying to me rather than trying to “read” their mind.”; 0.70 < *ω*_T1–T4_ < 0.85). The *Responsible Decision-Making competence questionnaire* is a unidimensional questionnaire composed of six items (e.g., “When I have a problem, I can think of alternative solutions.”; 0.71 < *ω*_T1–T4_ < 0.82). Items were evaluated on a 5-point scale (from 1 – *Never or hardly ever* to 5 – *Almost always or always*).

The *Positive and Negative Affect Schedule* (Watson et al., 1988; Portuguese version by Galinha and Pais-Ribeiro, 2005) was used to assess teachers perceived positive and negative affect. This questionnaire consists of 20 items organized in two scales: Positive affect (10 items, e.g., “Excited”; *ω*_T1_–_T4_ = 0.92) and Negative affect (10 items, e.g., “Afraid”; 0.90 < *ω*_T1–T4_ < 0.91). For each item participants were asked to rate how often they had felt each described emotion over a two-week period. The items were evaluated on a 5-point scale (from 1 – *Very slightly or not at all* to 5 – *Extremely*).

To measure how participants regulate their emotions in the face of different situations, the *Emotion Regulation Questionnaire* (Gross and John, 2003; Portuguese version by Vaz and Martins, 2008) was used. The questionnaire comprises 10 items organized in two scales: Cognitive reappraisal (6 items, e.g., “I control my emotions by changing the way I think about the situation I’m in.”; 0.87 < *ω*_T1–T4_ < 0.91) and Expressive suppression (4 items, e.g., “I keep my emotions to myself.”; 0.73 < *ω*_T1–T4_ < 0.81). Items were evaluated on a 7-point scale (from 1 – *Strongly disagree* to 7 – *Strongly agree*).

A *Self-care scale* (adapted from Vala et al., 2016) was used to assess teachers’ self-care behaviors. Four items were selected as indicators of self-care behaviors regarding physical activity, leisure, eating habits, and socialization with friends. The items were answered considering a three-months period (e.g., “Considering the last 3 months, how do you evaluate the care you have taken of yourself in terms of the practice of planned and regular physical exercise?”) and evaluated on a 5-point scale (from 1 – *Not at all satisfied* to 5 – *Totally satisfied*). The internal consistency for the total scale ranged from 0.71 to 0.80.

An indicator of subjective sleep quality retrieved from the *Pittsburgh Sleep Quality Index* (Buysse et al., 1989; Portuguese version by Rodrigues et al., 2014) was used to assess how teachers perceived their overall sleep quality in the previous month (i.e., “Considering the last month, how would you evaluate the overall quality of your sleep?”). The item was evaluated on a 4-point scale (from 1 – *Very bad* to 4 – *Very good*).

Teachers’ well-being was measured through the *Mental Health Continuum – Short Form* (Keyes et al., 2008; Portuguese version by Matos et al., 2010). The questionnaire comprises 14 items organized in three scales: Emotional well-being (3 items, e.g., “how often have you felt happy?”; 0.89 < ω_T1–T4_ < 0.92), Psychological well-being (6 items, e.g., “how often did you feel that you had experiences that challenged you to grow and become a better person?”; 0.91 < *ω*_T1–T4_ < 0.93), and Social well-being (5 items, e.g., “how often did you feel that you had something important to contribute to society?”; 0.82 < *ω*_T1–T4_ < 0.89). Items were evaluated considering the frequency of the described symptoms in the previous month on a 6-point scale (from 0 – *Never* to 5 – *Every day*).

An indicator of *Perceived occupational stress* (adapted from Kyriacou and Sutcliffe, 1978) was used to assess the degree to which teachers perceived their job as a stressful activity (i.e., “To what extent do you consider being a teacher as a stressful activity?”). The item was evaluated on a 5-point scale (from 1 – *Not at all stressful* to 5 – *Extremely stressful*).

Perceived experience of burnout symptoms was evaluated using the *Maslach’ Burnout Inventory - Educators Survey* (Maslach et al., 1996; Portuguese version by Marques-Pinto et al., 2005). The questionnaire is composed of 22 items organized in three dimensions: Emotional exhaustion (9 items, e.g., “I feel emotionally drained by my work.”; 0.91 < *ω*_T1–T4_ < 0.93), Depersonalization (5 items, e.g., “I feel students blame me for some of their problems.”; 0.85 < *ω*_T1–T4_ < 0.88), and Personal accomplishment (8 items, e.g., “I have accomplished many worthwhile things in this job.”; 0.83 < *ω*_T1–T4_ < 0.84). Items were evaluated on a 7-point scale (from 0 – *Never* to 6 – *Every day*).

The *Organizational Climate Description Questionnaire Revised for Elementary Schools* (Hoy and Clover, 1986; Portuguese version by Oliveira et al., 2022) was used to assess teachers’ perceptions of their school climate. This measure integrates 40 items organized in six scales. Three scales pertain to management behaviors at a leadership-level: Professional relationships management (10 items, e.g., “School coordinators listen and accept suggestions from teachers.”; 0.91 < *ω*_T1–T4_ < 0.92), Pedagogical tasks management (8 items, e.g., “School coordinators closely check the teaching practice.”; 0.79 < *ω*_T1–T4_ < 0.85), and Bureaucratic tasks management (5 items, e.g., “Administrative work is a burden at my school.”; 0.65 < *ω*_T1–T4_ < 0.75). The remaining three scales relate to teachers’ behavior within the school: Professional interactions among teachers (6 items, e.g., “Teachers help and support each other.”; 0.79 < *ω*_T1–T4_ < 0.89), Personal interactions among teachers (7 items, e.g., “Teachers socialize with each other regularly.”; 0.82 < *ω*_T1–T4_ < 0.85), and Dynamic of the teachers’ group (4 items, e.g., “Faculty meetings are useless.”; 0.64 < *ω*_T1–T4_ < 0.81). Items were evaluated on a 4-point scale (from 1 – *Rarely occurs* to 4 – *Very frequently occurs*).

#### Evaluation of the quality of the intervention implementation

To assess the quality of intervention implementation, systematic observation based on a *Synchronous Sessions Observation Grid* (SSOG) was performed by two trained independent observers. The SSOG was designed in the context of this study following Berkel et al.’s (2011, 2018) model and covers different parameters of the intervention implementation which are related to the program outcomes, namely facilitator behaviors (i.e., *fidelity* – one indicator concerning the concretization of session aims and another one relative to the staging of the activities on schedule; *quality* – one indicator related to the use of interactive teaching methods (e.g., brainstorming) and one indicator related to the clinical process skills (i.e., the ability to positively engage the participants in the session, to promote cohesion among the participants, and to present active listening and skillful feedback); and *adaptation* – one indicator reporting if any changes (modification, addition, or subtraction of contents) were performed in relation to the initial session plan) and the participants’ behaviors (i.e., *group responsiveness* – assessed through the participants’ active participation in the session). The fidelity-related indicators (e.g., “The session’s goals were accomplished.”) were rated on a 5-point scale varying from 1 – *None* to 5 – *All*. The clinical process skills (e.g., “The facilitator was capable of engaging the participants in the session.”) and active participation (i.e., “The participants actively participated in the proposed activities.”) items were rated on a 5-point scale varying from 1 – *Nothing* to 5 – *Very much*. Lastly, the items regarding interactive teaching methods (i.e., “Were interactive teaching methods used in the session?”) and adaptation (i.e., “During the implementation of the session, were any changes made in relation to the initial session plan?”) were rated on a dichotomous scale (0 – *No*, 1 – *Yes*), indicating whether or not interactive teaching methods and adaptations were used during the session. To estimate inter-rater reliability, a two-way mixed effects Intraclass Correlation Coefficient (ICC; absolute agreement) was performed. The ICC for inter-rater reliability was excellent (ICC = 0.98, 95% *CI* [0.97, 0.99]; Koo and Li, 2016). In addition to the SSOG, we have also evaluated: responsiveness of each participant, assessed individually for each participant through an indicator of participants’ active participation (rated on a 10-point scale ranging from 1 – *Did not participate at all* to 10 – *Participated constructively at all sessions*); attendance (i.e., measured through the number of sessions in which the teachers were present; maximum 10); and satisfaction (i.e., teachers evaluate their satisfaction throughout the training course on a 5-point scale ranging from 1 – *Not at all satisfied* to 5 – *Totally satisfied*; Berkel et al., 2011, 2018).

### Procedures

#### Data collection

Before we began recruiting participants, ethics approval for this study was obtained from the Scientific and Ethical Council of the Faculty of Psychology, University of Lisbon. School clusters were selected by convenience and authorization to conduct the study was obtained from the school principals. Elementary-school teachers within the school clusters and who complied with the eligibility criteria were invited to participate. Potential participants were contacted through their school’s training center and attended a meeting held by the first author where the study aims and participation procedures were described. Participants were self-selected.

As for the inclusion criteria, participants had to be teaching an elementary-school class (grades 1 to 4) during the school year in which the data collection occurred. Also, three exclusion criteria were considered: (1) teachers who did not have a class assigned; (2) teachers who were performing coordinating and/or supporting roles at the school-cluster; and (3) teachers who were responsible for teaching extracurricular activities, were not eligible for the study. As we followed a between-subjects design, after indicating their intention to enroll in the study and prior to pretest, the teachers were randomly assigned to either the EG or the CG within their school group. Written informed consents were obtained from the participants and data confidentiality and anonymity were ensured. Data collection protocols were identified with an alphanumeric code created by the participants themselves, allowing data to be crossed between the four data collection waves without revealing the participants’ identity. Following, the World Medical Association’s Declaration of Helsinki (WMA - World Medical Association, 2013), we ensured the participation was voluntary and the teachers could withdraw their participation at any time.

The *A+* intervention program was delivered in the form of a training course throughout 10 weekly-sessions and was accredited by the Pedagogical Scientific Council of Continuing Education (50-h for teachers’ career development). The training sessions were provided online for the participants within the EG across the three school clusters (i.e., 10 sessions × 3 groups), with support of the *Zoom* software and in collaboration with the schools’ training center. A trained and certified instructor, specializing in Educational Psychology, was responsible for delivering all the training sessions to the three intervention groups. The participants did not pay for the training course, however they were required to attend at least 2/3 of the sessions to receive the certificate. The teachers assigned to the waitlist CG did not have any intervention during this time but attended the *A+* after completion of the fourth data collection wave, in the same terms as the participants assigned to the EG.

To assess the quality of the intervention implementation, the SSOG was completed by a trained observer at all 30 training sessions. To ensure data validity and assess inter-rater reliability, a second trained observer simultaneously filled in the observation grid at 1/3 of the training sessions. The second observer was present at all 10 different sessions of the *A+*, although some may not have been delivered to the same group of participants.

To evaluate the *A+*’s efficacy, data was collected in four waves, simultaneously for the EG and the CG: prior to the intervention’s 1st training session (pretest held in September 2020), immediately after the last training session (posttest held in December 2020), 3 months after posttest (follow-up 1 held in March 2021), and 6 months after posttest (follow-up 2 held in June 2021). To guarantee social, cultural, and linguistic validity of the measures, we used Portuguese versions of all selected instruments and we ensured that their psychometric qualities had been previously studied with Portuguese samples. The data collection protocol was completed online through the *Qualtrics* platform^1^ and had an average response time of 30 min. All participants received a link to access the data collection protocol with the same instructions and at the same time, throughout the four data collection points. There were no missing values, since the software notified the participants of the need to complete their responses before submission. Also, participation was only registered when the full data protocol was completed. Regarding outliers’ detection, the analysis of the Q-Q plots depicted a tendency towards normal distribution of the data across the four data collection waves (i.e., |*z*| < 3; Kline, 2016). To reduce Social Desirability Bias (SDB), the anonymity and confidentiality of the responses were ensured, and a statement encouraging honesty was included at the beginning of the protocol (Larson, 2019).

#### Intervention

The *A+* is an online intervention program which sought to promote teachers’ SEC. As previously addressed, it builds on a prior study which tackles the planning of the intervention by means of a needs assessment within the intervention contexts and a pilot study to evaluate the social validity and efficacy of a trial version of the program (Oliveira et al., under revision). The SEL framework for teachers (Elias et al., 1997; Jennings and Greenberg, 2009; Durlak et al., 2015), the JD-R model (Demerouti et al., 2001; Schaufeli and Taris, 2014), and Collie’s (2020) Social and Emotional Competence School model ensured theoretical ground for the development of the *A+*’s contents and methodologies. While findings from previous studies on SEL interventions’ efficacy (Collie et al., 2012, 2018; Jennings et al., 2013, 2017, 2019; Carvalho et al., 2021) and on good practices for effective SEL (Durlak et al., 2010) and online (Hofmann, 2014; Beatty and Binnion, 2016; Kintu et al., 2017) interventions ensured the empirical ground for it.

More specifically, the SEL framework for teachers (Elias et al., 1997; Jennings and Greenberg, 2009; Durlak et al., 2015) informed on specific SEC domains that should be addressed (i.e., self-awareness, self-regulation, social awareness, relationship skills and responsible decision-making), and on specific behavioral and motivational strategies to promote the desired behavioral change (e.g., problem-focused coping strategies). The JD-R model (Demerouti et al., 2001; Schaufeli and Taris, 2014) provided support for the teacher-specific stressors and their associations with the variables that are expected to be impacted by this intervention (e.g., teacher burnout). Lastly, Collie’s (2020) Social and Emotional Competence School model depict an interactive process in which the development of SEC stem from an urge for autonomy, competence and relatedness needs satisfaction (Deci and Ryan, 1985), and that is influenced by the individual’s context and perceived social support. Hence, training groups were built within the same school cluster and different group activities were proposed to increase relatedness and teachers’ social support networks among peers.

Concerning its contents, the intervention program includes five components organized on the basis of stress-generating situations identified in the needs assessment: *Personal organization and time management* (seeking to promote teachers’ ability to set and achieve their goals, optimize their productivity, and to increase their adaptability skills in order to feel more comfortable in welcoming change and adjusting to new information or situations), *Emotional awareness and regulation* (aiming to promote teachers’ awareness of their individual characteristics, emotions and behaviors, and their ability to self-regulate their own emotions and consequent behaviors and decisions both in regular and challenging situations), *Conscious communication* (seeking to promote teachers’ open communication which contributes to their ability to build positive relationships and to collaborate with others), *Conflict management* (aiming to promote teachers’ ability to effectively prevent and manage conflict situations and negative social interactions and to work collaboratively towards finding common solutions while respecting others), and *Personal leadership* (seeking to promote teachers’ ability to make ethical and constructive decisions, evaluate and reflect on their behaviors, and to effectively solve problems). Table 2 depicts the structure, main contents, and examples of activities of the intervention program.

**Table 2:** Structure and contents of the *A*+ intervention program.

Module	SEC domains	Specific skills addressed	Main goal and example activity	Reflection topics
I – Personal organization and time management (Sessions 2 and 3)	Self-awareness and self-regulation	Accurate self-perception Goal setting and achieving Organizational skills Adaptability	*To set SMART goals:* Teachers perform a self-evaluation through a SWOT analysis. Based on these results, teachers are asked to identify specific social and/or emotional skills that require more investment. Then, teachers are guided to establish a SMART goal for one of these skills. *To increase work-life balance, through time organization:* Participants begin by identifying the main obstacles to their productivity which compromise their work-life balance. Next, through brainstorming, the group is guided to share strategies for personal organization and time management that they use and consider to be effective. Then, the facilitator presents a structured set of personal organization and time management strategies. Finally, teachers are asked to, for each obstacle previously identified, establish a strategy to test.	The impact of 21st century demands on teacher stress and burnout The importance of goal setting and strategic planning Importance of work-life balance for well-being, occupational health, and performance
II – Emotional awareness and regulation (Sessions 4 and 5)	Self-awareness and self-regulation	Emotional self-awareness Emotional and behavioral regulation Accurate self-perception Self-efficacy Optimism	*To develop emotional awareness:* The “*Discovering Emotions*” activity begins with a group reflection in which teachers reflect on their personal definition of emotion, emotion versus feeling, what emotions are for, the existence of good and bad emotions. Next, main thoughts are systematized by the facilitator. The facilitator then presents the theoretic content to answer the questions previously raised. The functions of the six primary emotions are explored as well as the physiological, cognitive, and behavioral dimensions of emotion. *To manage emotional symptoms:* During the exercise “*Physiology and State*”, teachers are asked to become aware of their physiological symptoms and emotional state while hearing the facilitator describe a positive and a negative situation. This is followed by a reflection on the importance of interpretation and thoughts in emotional states. Teachers are then guided, through changes in their body posture, to promote an emotional state of self-efficacy, strength and readiness.	Function of emotion and the importance of feeling the full emotional spectrum The role of thoughts and beliefs in the emotional experience Importance of emotional regulation
III – Conscious communication (Sessions 6 and 7)	Social awareness and relationship skills	Open communication Active listening Empathy Awareness of communication styles and non-verbal communication signs Organizational awareness	*To develop open communication and active listening skills:* The activity “*Wheel of Feelings*” consists of four rounds of increasing difficulty. In each round, participants are asked to, individually, answer a question while the remaining participants must actively listen to the answer. In the 1st round, participants must say whether they feel good or bad, at the present moment. In the 2nd round, they are asked to use an adjective to describe their physical state. In the 3rd round, participants must describe their emotional state with an adjective. In the last round, participants must explain why they are feeling any of the feelings expressed. In all rounds, participants can pass, and the response is voluntary. At the end, the discussion is open to the group, reflecting on the exercise.	The importance of verbal and non-verbal communication The importance of conscious communication in interpersonal relationships The role of growth mindset in ones’ attitude toward learning
IV – Conflict management (Sessions 7 and 8)	Social awareness and relationship skills	Taking perspective and appreciate diversity Being receptive to others’ feedback Respect for others Teamwork and collaboration	*To develop effective conflict management strategies:* In the exercise “*Managing a Difficult Conversation*”, teachers are asked to visualize two different situations they have experienced with the same person. One of them should represent a happy moment in which they felt connected to that person. The other situation should allude to a moment of conflict. The teachers should try to externally observe the two situations and analyze their behavior (e.g., emotional state, body posture, tone, communication style), identifying similarities and discrepancies. The facilitator then presents a set of conflict management strategies that teachers can implement from then on. Next, the participants are split into small groups and given a script which presents a conflict situation in which only one part of the conversation is presented. In small groups, the teachers should, applying the presented strategies, respond to the interlocutor of the script, in order to effectively manage the conversation. At the end, a spokesperson from each group presents the answer given by the group, thus, the large group tries to identify the implemented strategy and different possibilities. *To promote teamwork and collaboration:* In the “*Effective Communication at Work*” exercise, teachers are asked individually to identify a situation that they would like to see improved in their work context. The teachers then organize themselves into small groups and together come up with solutions/strategies that promote this change.	Assertiveness and respect for differences The importance of teamwork and collaboration toward goals
V – Personal leadership (Sessions 9 and 10)	Responsible decision making	Problem identification and situation analysis Problem-solving Behavior evaluation and reflection Making decisions	*To make decisions:* In this activity teachers are asked to establish a decision and then perform a best/worst analysis towards that decision. Teachers should anticipate the pros and cons of moving forward with that decision and of not moving forward. At the end, in a large group, teachers share their conclusions and experience with the exercise.	Consequences of decisions and behaviors

Session 1 corresponded to an introductory session where the participants introduced themselves to the group and shared their expectations with the intervention. Also in this 1^st^ session, the program structure was depicted, and the specific topics to be addressed were outlined. The platforms used and their specific resources and completion requirements were also presented in detail during this session. Session 7 appears in both modules III and IV since the topic of interpersonal conflicts was introduced in the 2nd part of the session.

Regarding its structure, the *A+* consisted of a total of 50 h of training, 25 of which were delivered in 10 weekly 2.5-h in-group synchronous sessions and 25 of which consisted in asynchronous training. The 25 h of synchronous training sessions were delivered through the *Zoom* software. The 25 h consisting of asynchronous training were supported by the *Moodle* platform. Each component of the *A+* had approximately 5 h of synchronous training (i.e., two training sessions). The intervention program followed the SAFE guidelines for interventions (i.e., sequenced training activities; active learning methods; focus on the development of SEC; and explicit SEL aims; Durlak et al., 2010). Thus, all the sessions included both expositive (e.g., introduction of new concepts, lectures) and active (e.g., brainstorming, role-playing) moments, and ended with a homework assignment (i.e., a weekly exercise related to the contents covered in the synchronous session and which aimed to help teachers implement the competences and skills addressed in their daily lives). Further description of the *A+* contents and structure, the specific procedural and methodological precautions for online interventions that were adopted, as well as illustrative examples of the *Zoom* and *Moodle* elements are depicted in the Supplementary materials.

#### Data analysis

Reliability of the measures was studied through internal consistency calculated with the coefficient omega (ω; Peters, 2014). Internal consistency was considered adequate for values above a minimum of.50, and good when scores were equal to or above.70 (Crutzen and Peters, 2017). Analyses were computed using the *effects* (effect displays for linear, generalized linear, and other models; Fox and Weisberg, 2019), *effectsize* (indices of effect size and standardized parameters; Ben-Shachar et al., 2020), *lme4* (linear mixed-effects models using ‘Eigen’ and S4; Bates et al., 2015), *robustbase* (basic robust statistics; Maechler et al., 2022), *robustlmm* (robust linear mixed effects models; Koller, 2016), *ufs* (a collection of utilities; Peters and Gruijters, 2021), and *WRS2* (collection of robust statistical methods; Mair and Wilcox, 2020) packages designed for R environment (R Core Team, 2019).

##### Intervention’s efficacy

First, as the present study aimed to evaluate the impact of the *A+* on the outcome variables, baseline analyses were performed to explore whether there were any initial differences between the school clusters, namely regarding the sociodemographic, SEC, and contextual variables. Due to the sample size within the clusters, all the analyses were performed using robust statistics. Thus, robust one-way *ANOVA*s based on trimmed means (20% trimming level) and *post hoc* tests were performed, at baseline, to compare sociodemographic, organizational climate and SEC variables between the school clusters. Then, to address our research question and anticipating a relationship between contextual and proximal dependent variables, Spearman correlations were computed to evaluate the association between organizational climate and SEC, and robust linear regression models were performed to evaluate whether perceived organizational climate predicted teachers’ SEC at baseline. Following Cohen’s (1988) criteria, correlation values around 0.10, 0.30, and 0.50 illustrate small, moderate, and large associations, respectively. To test our research hypotheses, the data analyses were performed within school clusters. Thus, robust one-way *ANOVA*s were also computed to analyze baseline differences across sociodemographic, organizational climate and SEC variables between the treatment conditions within school clusters. To account for family-wise error rate, the Holm-Bonferroni method was used for multiple comparisons (Mair and Wilcox, 2020; Maechler et al., 2022). Lastly, to test our research hypotheses, robust linear mixed effects models with 95% confidence intervals (*CI*) with bootstrap for the estimates were performed within school clusters to test for the intervention effects on the outcome variables. Interaction effects between the treatment conditions and the four data collection waves were estimated while controlling for the variables proving to be significant at baseline. To evaluate the magnitude of the findings, effect sizes with 95% confidence intervals with bootstrap were calculated for robust one-way *ANOVA*s using the Partial Eta Squared ($\eta^{2}$). For robust regression and mixed effects models, effect sizes were measured using Cohen’s *f* (*f*^2^). Estimates were considered significant whenever the 95% bootstrapped confidence intervals did not include 0.

##### Quality of the intervention implementation

Regarding the indicators of quality of the intervention’s implementation and their impacts on the program outcomes, fidelity, quality, and group responsiveness indicators were computed. Total indicators of fidelity, quality, and group responsiveness were calculated through the average evaluation across the 10 synchronous sessions. Partial indicators of fidelity, quality, and group responsiveness allowing a more refined understanding of the implementation processes were estimated through the average evaluation of the synchronous sessions within each program component. Additionally, robust linear regression models were computed within the school clusters following the previously stated analytical strategy (see Section “Intervention’s efficacy”) to evaluate if participants’ individual responsiveness predicted their perceived SEC at posttest.

## Results

### Baseline analysis

#### Comparisons between school clusters regarding demographic, contextual and proximal dependent variables

Regarding the demographic variables, statistically significant differences between the school clusters were found for teachers’ years of teaching experience (*F*(2, 28.1) = 7.62, $\eta^{2}$ = 0.52, 95% *CI* [0.28, 0.73]), with participants from Cluster B presenting longer years of teaching experience than teachers from Cluster A ($\overset{⌢}{\Psi }$ = −10.83, 95% *CI* [−17.92, −3.74]) and from Cluster C ($\overset{⌢}{\Psi }$ = 7.74, 95% *CI* [0.38, 15.10]). No differences were found regarding teachers’ age and previous attendance at social and emotional learning interventions.

Concerning perceived organizational climate, robust *ANOVA*s depicted statistically significant differences between the school clusters for two dimensions: Professional relationships management (*F*(2, 30.59) = 9.10, $\eta^{2}$ = 0.54, 95% *CI* [0.34, 0.78]) and Personal interactions among teachers (*F*(2, 26.43) = 4.01,$\eta^{2}$ = 0.40, 95% *CI* [0.20, 0.67]). Post-hoc comparisons revealed that Cluster B presented higher perceptions of Professional relationships management than Cluster A ($\overset{⌢}{\Psi }$ = −0.50, 95% *CI* [−0.82, −0.19]) and Cluster C ($\overset{⌢}{\Psi }$ = 0.45, 95% *CI* [0.08, 0.82]). Cluster B also presented higher perceptions of Personal interactions among teachers than Cluster C ($\overset{⌢}{\Psi }$ = 0.31, 95% *CI* [0.01, 0.64]). No statistically significant differences were found between clusters A and C.

No differences were found regarding teachers’ SEC between the school clusters.

#### Relations between organizational climate and proximal dependent variables

The correlation analysis indicated that, at pretest, all the organizational climate dimensions presented statistically significant associations with some of the teachers’ assessed SEC variables, except for Professional relationships management (Supplementary Table S1). A subsequent robust linear regression analysis revealed that, at baseline, Positive affect was positively predicted by Bureaucratic tasks management (*B* = 0.30, *SE* = 0.11, 95% *CI* [0.07, 0.53], *R*^2^ = 0.06, *f*^2^ = 0.27), and Expressive suppression was positively predicted by Pedagogical tasks management (*B* = 0.66, *SE* = 0.32, 95% *CI* [0.01, 1.30], *R*^2^ = 0.11, *f*^2^ = 0.28). Moreover, Professional interactions among teachers was a positive predictor of perceived Self-regulation (*B* = 0.46, *SE* = 0.15, 95% *CI* [0.15, 0.76], *R*^2^ = 0.07, *f*^2^ = 0.54), Positive relationship (*B* = 0.41, *SE* = 0.19, 95% *CI* [0.02, 0.79], *R*^2^ = 0.13, *f*^2^ = 0.53), and Conflict management (*B* = 0.50, *SE* = 0.15, 95% *CI* [0.20, 0.79], *R*^2^ = 0.28, *f*^2^ = 0.63) skills. Conflict management was also negatively predicted by Personal interactions among teachers (*B* = −0.39, *SE* = 0.10, 95% *CI* [−0.59, −0.19], *R*^2^ = 0.28, *f*^2^ = −0.47).

These analyses supported the relationship between contextual variables and the proximal dependent variables, with perceived organizational climate predicting teachers’ SEC. Additionally, baseline comparisons between the school clusters also revealed statistically significant differences across perceived organizational climate. Thus, analyses of the intervention program’s efficacy and the quality of the intervention’s implementation were performed within the school clusters.

#### Comparisons between treatment conditions within school clusters regarding demographic and outcome variables

No differences were found between the treatment conditions at baseline for Cluster A. Within Cluster B, no differences were found regarding demographic variables, however the EG evidenced a higher positive relationship (*F*(1, 13.96) = 4.85, *p* = 0.045, $\eta^{2}$ = 0.48, 95% *CI* [0.09, 0.93]) and responsible decision-making (*F*(1, 11.80) = 5.10, *p* = 0.044, $\eta^{2}$ = 0.49, 95% *CI* [0.03, 1.06]) skills at baseline compared to the CG. As for Cluster C, the teachers in the EG revealed higher expressive suppression (*F*(1, 21.71) = 8.69, $\eta^{2}$ = 0.66, 95% *CI* [0.34, 0.93]) and lower positive relationship skills (*F*(1, 22.46) = 5.61, $\eta^{2}$ = 0.46, 95% *CI* [0.03, 0.83]) than the teachers in the CG. No differences were found regarding demographic variables in this school cluster.

### Intervention’s efficacy

In order to help determine the direction of change, Supplementary Tables S2–S4 in the Supplementary material present the means and standard deviations for all the dependent variables for the EG and CG within the school clusters and across time.

#### Cluster A

**Figure 3:**
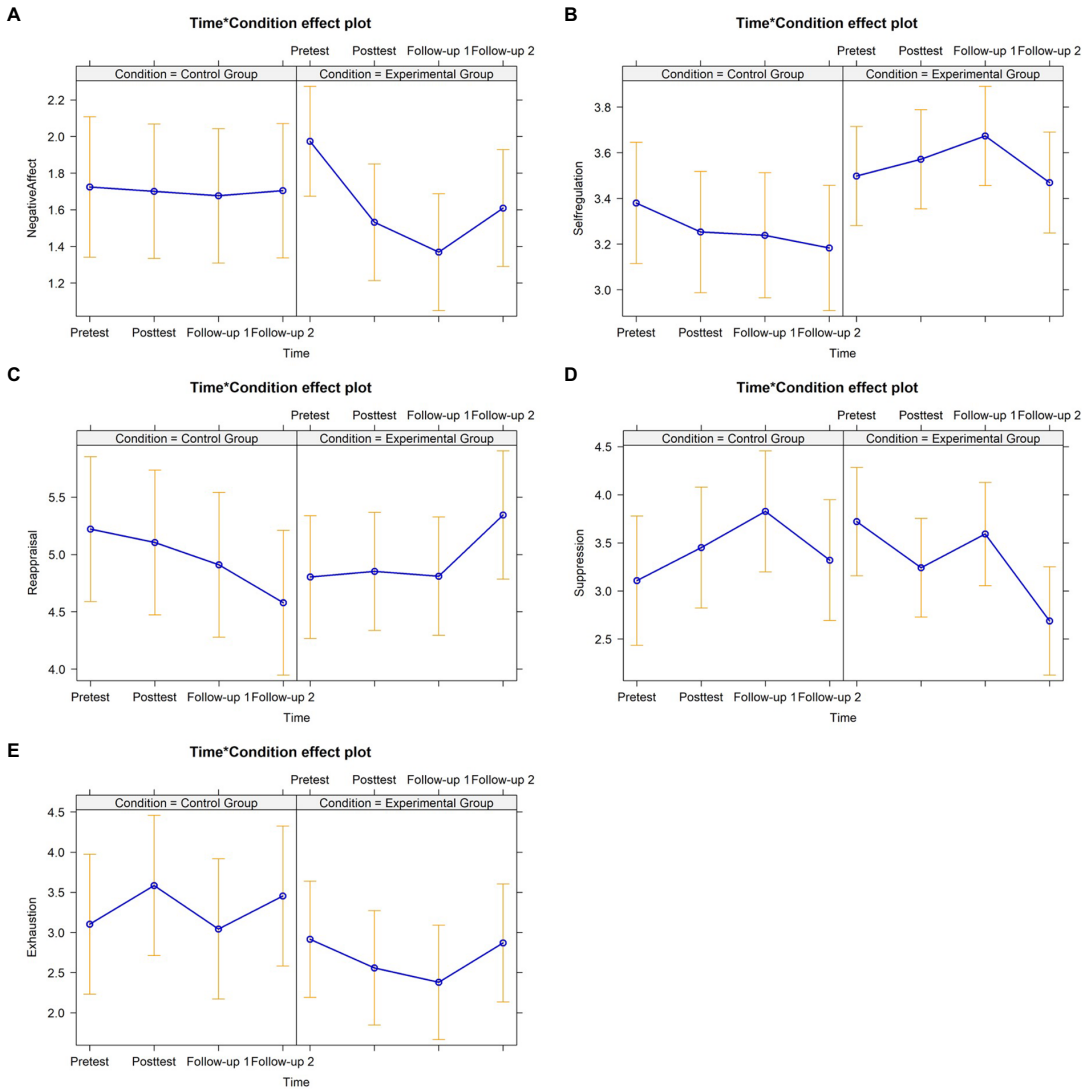
Graphical representation of interaction effects (Time*Treatment Condition) on proximal and distal dependent variables – Cluster A. **(A)** Negative affect depicting a significant effect at follow-up-1 (*B* = −0.56, *SE* = 0.26, 95% *CI* [−1.06, −0.06], *f*^2^ = −0.91). **(B)** Self-regulation skills depicting a significant effect at follow-up-1 (*B* = 0.32, *SE* = 0.14, 95% *CI* [0.04, 0.59], *f*^2^ = 0.78). **(C)** Cognitive reappraisal depicting a significant effect at follow-up-2 (*B* = 1.18, *SE* = 0.51, 95% *CI* [0.18, 2.18], *f*^2^ = 1.38). **(D)** Expressive suppression depicting a significant effect at follow-up-2 (*B* = −1.25, *SE* = 0.61, 95% *CI* [−2.44, −0.05], *f*^2^ = −1.38). **(E)** Emotional exhaustion symptoms depicting a significant effect at posttest (*B* = −0.84, *SE* = 0.40, 95% *CI* [−1.62, −0.06], *f*^2^ = −0.71).

The analysis of baseline differences between the treatment conditions showed no differences across the demographic and outcome variables. Robust linear mixed effects models evidenced interaction effects on teachers’ SEC and burnout symptoms, favoring teachers from the EG. Regarding SEC, the teachers who attended the *A+* program showed a decrease in negative affect and an increase in self-regulation skills at follow-up 1, and an increase of cognitive reappraisal and decrease in expressive suppression at follow-up 2. Concerning the distal dependent variables assessed, the teachers from the EG reported a decrease of emotional exhaustion symptoms at posttest. A graphical representation of the interaction effects is presented in Figure 3.

#### Cluster B

**Figure 4:**
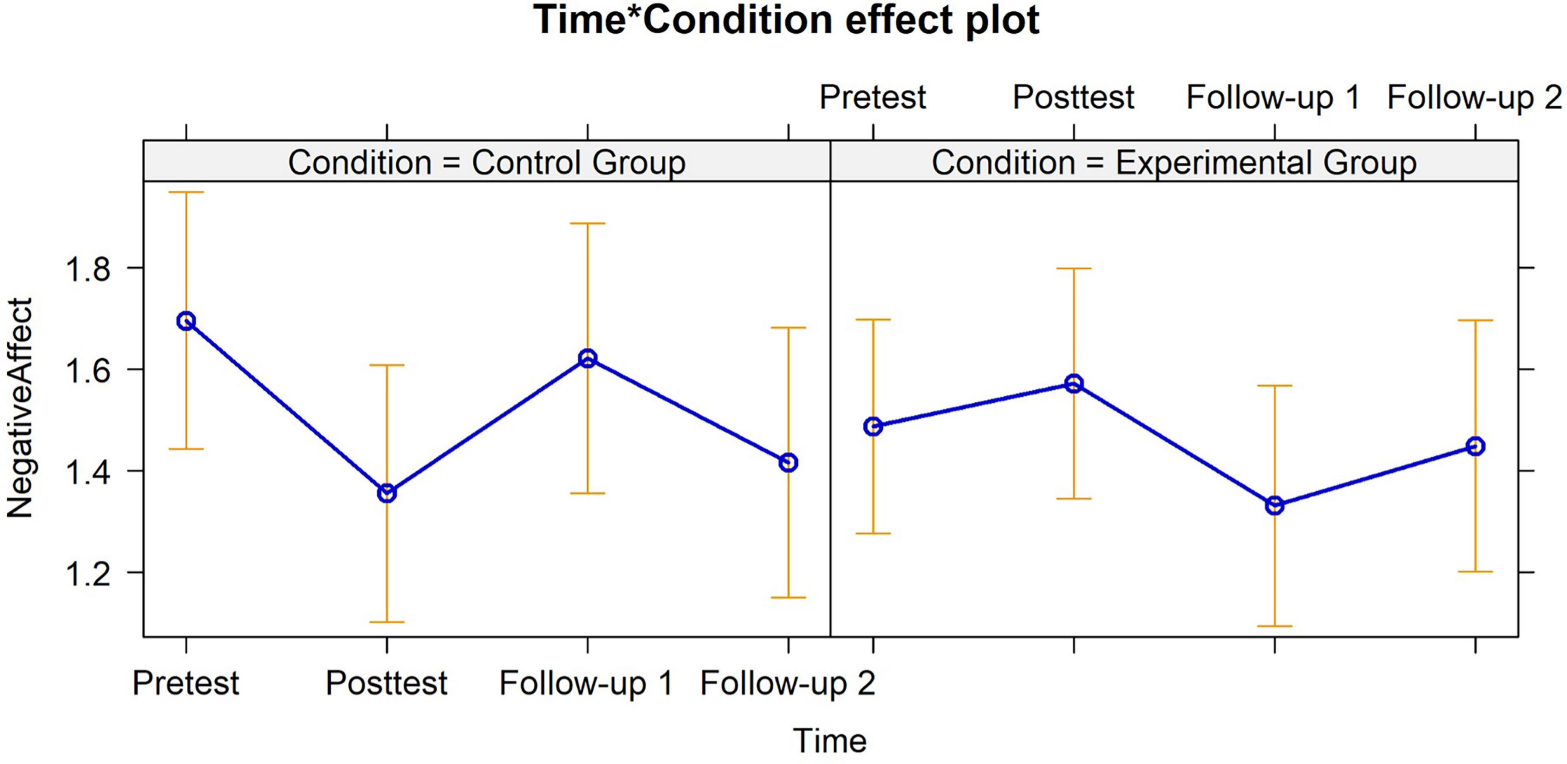
Graphical representation of interaction effects (Time*Treatment Condition) on proximal and distal dependent variables – Cluster B. Negative affect depicting a significant effect at posttest (*B* = 0.43, *SE* = 0.20, 95% *CI* [0.04, 0.82], *f*^2^ = 0.95).

After controlling for significant baseline variables (i.e., positive relationship and responsible decision-making), the results showed increased negative affect within the EG at posttest (Figure 4). No other interaction effects were reported regarding Cluster B.

#### Cluster C

**Figure 5:**
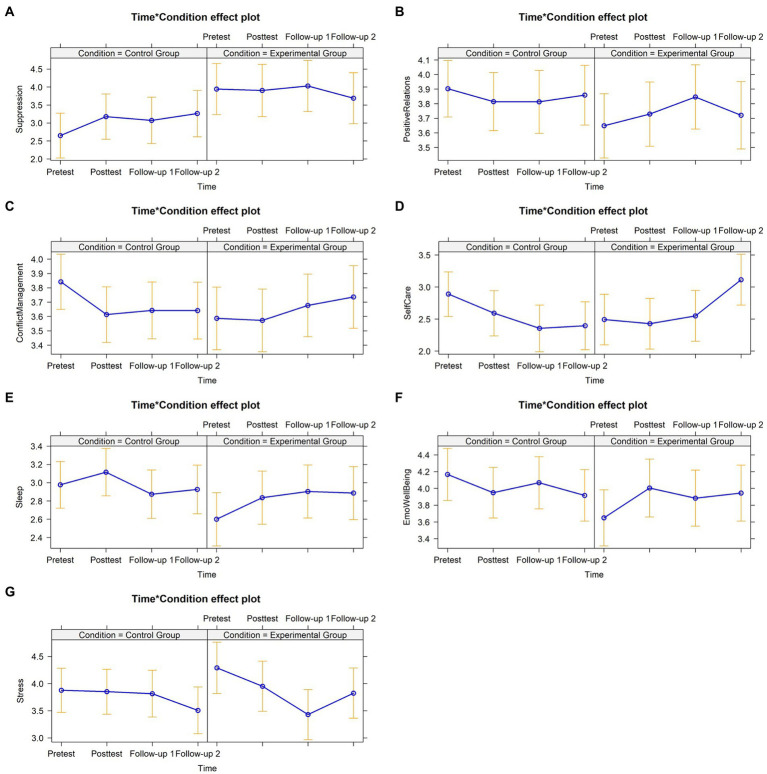
Graphical representation of interaction effects (Time*Treatment Condition) on proximal and distal dependent variables – Cluster C. **(A)** Expressive suppression depicting a significant effect at follow-up 2 (*B* = −0.93, *SE* = 0.38, 95% *CI* [−1.66, −0.19], *f*^2^ = −0.69). **(B)** Positive relationship skills depicting a significant effect at follow-up-1 (*B* = 0.32, *SE* = 0.13, 95% *CI* [0.07, 0.58], *f*^2^ = 0.74). **(C)** Conflict management skills depicting a significant effect at posttest (*B* = 0.21, *SE* = 0.11, 95% *CI* [0.03, 0.43], *f*^2^ = 0.48), follow-up 1 (*B* = 0.29, *SE* = 0.11, 95% *CI* [0.07, 0.51], *f*^2^ = 0.65) and follow-up 2 (*B* = 0.35, *SE* = 0.11, 95% *CI* [0.13, 0.56], *f*^2^ = 0.78). **(D)** Self-care practices depicting a significant effect at follow-up 1 (*B* = 0.59, *SE* = 0.24, 95% *CI* [0.13, 1.06], *f*^2^ = 0.67) and follow-up 2 (*B* = 1.12, *SE* = 0.24, 95% *CI* [0.64, 1.59], *f*^2^ = 1.27). **(E)** Sleep quality depicting a significant effect at follow-up 1 (*B* = 0.41, *SE* = 0.17, 95% *CI* [0.08, 0.74], *f*^2^ = 0.60) and follow-up 2 (*B* = 0.34, *SE* = 0.17, 95% *CI* [0.01, 0.67], *f*^2^ = 0.50). **(F)** Emotional well-being depicting a significant effect at posttest (*B* = 0.57, *SE* = 0.21, 95% *CI* [0.16, 0.99], *f*^2^ = 0.78) and follow-up 2 (*B* = 0.54, *SE* = 0.21, 95% *CI* [0.13, 0.95], *f*^2^ = 0.74). **(G)** Occupational stress depicting a significant effect at follow-up 1 (*B* = −0.80, *SE* = 0.34, 95% *CI* [−1.46, −0.13], *f*^2^ = −0.83).

After controlling for significant baseline variables (i.e., expressive suppression and positive relationship skills), the findings showed statistically significant interaction effects favoring the EG across teachers’ perceived SEC, self-care, well-being, and occupational stress (Figure 5). Concerning the proximal dependent variables, the teachers who had attended the program presented: a decrease in expressive suppression at follow-up 2, an increase in positive relationship at follow-up 1, and an increase in conflict management skills at posttest, follow-up 1 and follow-up 2. Regarding the distal dependent variables measured, the teachers who had attended the *A+* evidenced higher self-care practices at follow-up 1 and follow-up 2. Moreover, the teachers from the EG also showed improved sleep quality at follow-up 1 and follow-up 2, emotional well-being at posttest and follow-up 2, and a reduction of perceived occupational stress intensity at follow-up 1.

### Quality of the intervention implementation

The means and standard deviations of the total and partial indicators of intervention implementation quality across the school clusters are depicted in Table 3. No statistically significant differences regarding the SSOG’s quality of implementation’s indicators or the individual participants’ responsiveness were found between the school clusters. Assessment of Interactive teaching methods revealed that active methodologies (e.g., individual and group reflections, role-playing, written exercises, brainstorming) were present at all sessions throughout the school clusters, justifying the absence of variability found in the table (*M* = 1.00, *SD* = 0.00). Also, the facilitator was perceived to present clinical process skills consistently throughout the sessions and across the training groups (*M* = 5.00, *SD* = 0.00, except for the Cluster A’s 1st training component). Specifically, the facilitator was perceived to be able to positively engage the participants in the session, to promote cohesion among the participants, and to present active listening and skillful feedback. Regarding the participants’ own evaluation of the quality of implementation, Satisfaction with the program was high, particularly for the Cluster C. Also, all clusters showed a good Attendance to the sessions, with Group B showing the highest rate of presence.

**Table 3:** Mean and standard deviation for the dimensions of program implementation quality in accordance with the integrated model of program implementation by Berkel et al. (2011, 2018).

	Facilitator behaviors				Participant behaviors		
	Fidelity	Quality		Adaptation	Group responsiveness		
		Interactive teaching methods	Clinical process		Active participation	Satisfaction	Attendance
	*M*(SD)	*M* (SD)	*M* (SD)	*M* (SD)	*M* (SD)	*M* (SD)	*M* (SD)
*Cluster A*							
Total	4.88 (0.21)	1.00 (0.00)	4.98 (0.05)	0.25 (0.35)	4.70 (0.42)	4.50 (0.80)	9.08 (1.04)
Personal organization and time management	4.92 (0.14)	1.00 (0.00)	4.94 (0.10)	0.50 (0.50)	5.00 (0.00)	–	
Emotional awareness and regulation	5.00 (0.00)	1.00 (0.00)	5.00 (0.00)	0.00 (0.00)	4.50 (0.71)		
Conscious communication	4.75 (0.35)	1.00 (0.00)	5.00 (0.00)	0.25 (0.35)	5.00 (0.00)		
Conflict management	4.50 (0.00)	1.00 (0.00)	5.00 (0.00)	0.50 (0.00)	4.50 (0.00)		
Personal leadership	5.00 (0.00)	1.00 (0.00)	5.00 (0.00)	0.00 (0.00)	4.25 (0.35)		
*Cluster B*							
Total	4.85 (0.34)	1.00 (0.00)	5.00 (0.00)	0.60 (0.52)	5.00 (0.00)	4.67 (0.78)	9.33 (0.81)
Personal organization and time management	5.00 (0.00)	1.00 (0.00)	5.00 (0.00)	0.67 (0.58)	5.00 (0.00)	–	
Emotional awareness and regulation	5.00 (0.00)	1.00 (0.00)	5.00 (0.00)	0.50 (0.71)	5.00 (0.00)		
Conscious communication	4.50 (0.72)	1.00 (0.00)	5.00 (0.00)	1.00 (0.00)	5.00 (0.00)		
Conflict management	4.50 (0.00)	1.00 (0.00)	5.00 (0.00)	1.00 (0.00)	5.00 (0.00)		
Personal leadership	5.00 (0.00)	1.00 (0.00)	5.00 (0.00)	0.00 (0.00)	5.00 (0.00)		
*Cluster C*							
Total	4.90 (0.21)	1.00 (0.00)	5.00 (0.00)	0.40 (0.52)	4.80 (0.42)	4.81 (0.54)	8.81 (0.87)
Personal organization and time management	5.00 (0.00)	1.00 (0.00)	5.00 (0.00)	0.67 (0.58)	5.00 (0.00)	–	
Emotional awareness and regulation	5.00 (0.00)	1.00 (0.00)	5.00 (0.00)	0.00 (0.00)	5.00 (0.00)		
Conscious communication	4.75 (0.35)	1.00 (0.00)	5.00 (0.00)	0.50 (0.71)	4.50 (0.71)		
Conflict management	4.50 (0.00)	1.00 (0.00)	5.00 (0.00)	1.00 (0.00)	5.00 (0.00)		
Personal leadership	5.00 (0.00)	1.00 (0.00)	5.00 (0.00)	0.00 (0.00)	4.50 (0.71)		

Rating scales ranged from: 1 to 5 with regard to the Fidelity, Clinical process, Active participation, and Satisfaction indicators; 0 (i.e., absence) to 1 (i.e., presence) with regards to the Interactive teaching methods and Adaptation indicators; and from 1 to 10 (synchronous sessions) with regard to the Attendance indicator.

#### Cluster A

An analysis of both the facilitator and participants’ behaviors revealed good quality of intervention implementation results for all the program components. Some adaptations were made in the Personal organization and time management (namely, at the group’s request, no break was held), Conscious communication (namely, in Session 6, the duration of the activity “Wheel of Feelings” was extended from the initial plan, motivated by the pertinent sharing and reflections of the participants. Thus, the introduction of non-verbal communication elements was exposed by the facilitator, and the initially planned brainstorming was suppressed), and Conflict management modules (namely, toward the group’s interest in the group exercise of conflict management, the time invested in this activity was extended from the initial plan). As a result of these adaptations, fidelity was rated relatively lower for the same modules, particularly the Conscious communication and Conflict management components where the alterations to the initial plan were most impactful. The group responsiveness was good throughout the program, but it was relatively lower in the Personal leadership module compared to the other components. Within Cluster A, robust linear regression models revealed that participants’ individual responsiveness (*M* = 8.19, *SD* = 1.37, *Median* = 8.67, 1st quartile = 7.08, 3rd quartile = 9.25, *Interquartile range =* 2.17) did not predict the proximal dependent variables at posttest.

#### Cluster B

The indicators supported the good quality of the intervention’s implementation. All the program components were subject to adaptations except for the Personal leadership module, and fidelity was rated relatively lower for the Conscious communication and Conflict management components. Particularly, for this school cluster most adaptations regarded the suppression of the break foreseen in the plan, in order to allow the group’s reflections and sharing to be accommodated while ensuring that fidelity was not compromised. However, following the experience of Cluster A, the duration of the activity “Wheel of Feelings” and the group exercise of conflict management required more time than anticipated, thus impacting the fidelity of the Conscious communication and Conflict management modules. For Cluster B, group responsiveness was excellent and did not vary across the intervention’s implementation. Nevertheless, within this cluster, participants’ individual responsiveness (*M* = 8.25, *SD* = 1.22, *Median* = 8.17, 1st quartile = 7.00, 3rd quartile = 9.58, *Interquartile range =* 2.58) predicted proximal program outcomes for teachers in the EG. The teachers who had attended the *A+* and were more responsive in the synchronous sessions reported higher positive affect (*B* = 0.40, *SE* = 0.10, 95% *CI* [0.17, 0.63], *R*^2^ = 0.44, *f*^2^ = 0.60), self-regulation (*B* = 0.24, *SE* = 0.09, 95% *CI* [0.02, 0.45], *R*^2^ = 0.50, *f*^2^ = 0.53), and responsible decision-making skills (*B* = 0.17, *SE* = 0.07, *R*^2^ = 0.21, 95% *CI* [0.01, 0.32], *f*^2^ = 0.60), and lower negative affect (*B* = −0.13, *SE* = 0.04, 95% *CI* [−0.23, −0.04], *R*^2^ = 0.68, *f*^2^ = −0.26) and expressive suppression (*B* = −0.53, *SE* = 0.22, 95% *CI* [−1.02, −0.04], *R*^2^ = 0.54, *f*^2^ = −0.55) at posttest.

#### Cluster C

Within Cluster C, an analysis of the quality of the intervention’s implementation parameters showed good indicators. Following the experience of the other training groups, adaptations were relatively higher for the Personal organization and time management, Conscious communication and Conflict management components. The adaptations particularly concerned the adjustment of the duration of the activities with active methodologies, to minimize the impacts on the fidelity of the sessions. Nevertheless, as occurred with the other clusters, fidelity was rated lower for the Conscious communication and Conflict management modules. Within Cluster C, the group was less responsive in the Conscious communication and Personal leadership components, requiring from the facilitator a higher effort to stimulate participants’ active participation. Regarding the participants’ individual responsiveness (*M* = 7.88, *SD* = 1.41, *Median* = 8.00, 1st quartile = 6.33, 3rd quartile = 9.17, *Interquartile range =* 2.84), it predicted proximal program outcomes in this intervention group, with the more responsive teachers reporting, higher cognitive reappraisal (*B* = 0.33, *SE* = 0.14, 95% *CI* [0.02, 0.64], *R*^2^ = 0.17, *f*^2^ = 0.49) at posttest.

## Discussion

### Summary of the main results

This study aimed to assess the *A+* efficacy on teachers’ proximal (i.e., SEC) and distal (i.e., self-care practices, sleep quality, well-being, occupational stress, and burnout symptoms) outcomes. Furthermore, adding to the literature in the field, it also sought to assess the role of implementation quality and contextual variables on the program’s outcomes. Overall, the findings revealed good indicators of the *A+*’s efficacy in promoting teachers’ resources to respond to previously identified job demands and increasing occupational health and well-being symptoms. Nevertheless, the results substantially differed across the school-clusters and were not stable across time, thus requiring further reflection.

#### Findings on intervention’s efficacy

In line with prior research (Collie, 2017; Taris et al., 2017), our findings reinforce the importance of considering contextual variables when designing, implementing, and assessing SEL interventions. In this study, organizational climate appeared to predict teachers’ SEC which, following previous research, can interfere with teachers’ SEL needs (Jennings and Greenberg, 2009; Collie, 2017). In keeping with prior literature, Cluster B which perceived a healthier organizational climate (namely through the organizational support/democratic leadership and the maintenance of cohesive and strong social relationships with colleagues), also revealed higher protection factors (namely higher means of SEC at baseline). Conversely, clusters A and C which perceived a more closed and unhealthier organizational climate, reported not only lower means of SEC at baseline (across all the assessed variables), but also perceived lower self-care practices, social well-being, and personal accomplishment, and higher occupational stress and emotional exhaustion symptoms, in comparison to Cluster B. Thus, in answer to our research question (*Q1*), indeed, a positive organizational climate seems to promote teachers’ SEC and may, accordingly, impact teachers’ perceived SEL needs and professional demands and resources. It seems, therefore, important to take contextual variables into account due to their direct impact on teachers’ personal resources to effectively respond to job demands. Moreover, these dissimilarities between working contexts, also seem to interfere with SEL intervention’s efficacy.

In this study, mixed results were found regarding the *A+*’s efficacy which deserve attention. Not only the clusters A and C (which described an unhealthier organizational climate) appear to have benefited more from the intervention program, but also the impacts of the *A+* differed between these two contexts. Therefore, it seems necessary to interpret the results considering the specificities of the different contexts, previously depicted in the section “Participants”.

With regards to Cluster A, the *A+* was particularly effective in promoting teachers’ resources associated with emotional regulation (as data suggests an increase in self-regulation skills and use of cognitive reappraisal, and a decrease in the use of expressive suppression). This was, in fact, the dimension in which teachers within this context appear to feel less competent, leading to the experience of negative emotions and related symptoms (such as, emotional exhaustion). Interestingly, not only did the emotion regulation skills appear to change the most post-intervention but, additionally, the EG teachers also reported a decrease in negative affect and emotional exhaustion symptoms. Therefore, regarding Cluster A, hypotheses 1 and 2b were partially sustained, while hypothesis 2a was not confirmed.

Concerning Cluster B, the *A+* seems to have not been effective in promoting teachers’ SEC nor their occupational health. Moreover, within this cluster, the results indicated an unexpected increase of negative affect for the teachers who had benefitted from the intervention, at posttest. However, even though emotional regulation skills emerged as the main challenge for these teachers at the needs assessment, this was, of the three, the school cluster which described fewer needs/risk factors. Moreover, the findings suggested that: not only there were no significant differences in the baseline level of SEC between school clusters, but there was also a tendency for teachers from Cluster B to present higher mean scores towards these skills. Thus, as far as Cluster B is concerned, there are different explanatory hypotheses that may have been at the origin of these results. On one hand, it is possible that a ceiling effect has occurred in view of which the contents of the intervention were not effective in further contributing to the development of these teachers’ SEC. Also, in view of these findings, it is possible that teachers in Cluster B did not perceive a need for behavioral change given their low perception of risk factors (Schwarzer, 2016). Furthermore, the apparently perverse increase in teachers’ negative affect at posttest (which was not maintained across time) can also relate to the very positive perception of these teachers’ working context. More specifically, as aforementioned (*vide* section “Participants”), this school-cluster is characterized by strong interpersonal relations. Therefore, this result may be reflecting teachers’ resistance to change, possibly driven by the uncertainty of how these new behaviors would be received by their peers. Following the *Self-determination theory* (Deci and Ryan, 1985), human behavior is motivated by relatedness, which regards to the feeling of being close to others/significant part of a social group. Also, in accordance with the *Theory of planned behavior* (Ajzen, 2005) an intention to behave depends on the individuals’ subjective norms (i.e., the belief on how a behavior would be approved/disapproved by the group). Thus, as teachers from the Cluster B valued the interpersonal relations with their colleagues, assessed their work environment to be positive and presented fewer demands, group allegiance and outcome expectancies toward behavior change may have affected the results. Still, it should not be disregarded that participating in this intervention program may have increased teachers’ workload (namely since at posttest, teachers from the EG were completing assignments to conclude their certification, whilst teachers from the CG did not have this additional task) thus interfering with teachers’ affect and stress at posttest (Granziera et al., 2021). Therefore, regarding Cluster B, none of the hypotheses were confirmed.

Finally, for Cluster C, the *A+* seems to have been particularly effective in promoting teachers’ positive relationship and conflict management skills, and in decreasing teachers’ use of expressive suppression. As described in the participants’ characterization, this was the context that presented the most personal risk factors (particularly related to interpersonal relationship and emotional regulation), leading to demotivation and negative emotions. Hence, findings suggest that the *A+* was effective in increasing teachers’ resources in the dimensions where teachers perceived to have greater needs. Moreover, following the *Theory of planned behavior* (Ajzen, 2005), we know that subjective norms are important to determine individual’s behavioral intentions. Also, in this school cluster, social support between peers emerged as the most valued resource. Then, relatedness is important for these teachers (Deci and Ryan, 1985). However, contrary to what was observed for Cluster B, in this school cluster there was a high identification of professional demands both at a personal and at the working context levels. Thus, the perceived need for behavioral change would be higher for this group (Schwarzer, 2016). Additionally, as the intervention groups were built within the same school cluster to facilitate the identification and resolution of specific problems within the context, it is possible that this group connection has contributed to the faster and more permanent development of interpersonal skills (i.e., positive relationship and conflict management skills) in these teachers. With teachers feeling an increase in the resources perceived to be lacking, the results obtained in the distal variables (i.e., self-care practices, sleep quality, well-being, and reduced stress) are in line with what is expected and suggested by the literature (Jennings et al., 2013, 2017, 2019; Harris et al., 2016; Oliveira et al., 2021a). Therefore, regarding Cluster C all three hypotheses were partially sustained.

Taken together, these findings suggest that the intervention was effective in addressing the main needs identified by teachers within the different school clusters. However, the findings also point to sleeper effects found in SEC (with most effects emerging only in follow-up 1 and follow-up 2) and the fragile stability of the proximal and distal results across time and within the different school clusters. This suggests the possible need to increase the duration of the intervention program and/or to develop complementary booster sessions. In line with prior literature, duration (i.e., distance between the 1^st^ and last training sessions) above dosage (i.e., number of effective training hours) appears to impact program outcomes (Oliveira et al., 2021b). Furthermore, particularly because self-report measures were used in the present study, which entail a change in the perception of behaviors, the findings may be more sensitive to the participant’s perception and, therefore, require more time to reflect changes. As teachers already automatized behavior patterns regarding social and emotional skills (even those that are ineffective and unhealthy, e.g., regulating emotions through expressive suppression), they might require a longer period to experiment new behaviors, evaluate the results, (re)adapt or maintain the change. In accordance with the *Health Action Process Approach* framework, behavior change, particularly when related to crystallized behaviors, is demanding and requires several factors in addition to the intention to change (e.g., risk perception; action, maintenance and recovery self-efficacy; outcome expectancies; action control) (Schwarzer, 2016), thus requiring time for the changes to be expressed in the self-report questionnaires.

Additionally, most of the interaction effects found resulted from not only gains in the EG in the expected direction, but also CG reductions in perceived SEC, self-care practices, sleep quality, and well-being, and the enhancement of burnout symptoms. Also, the majority of interaction effects were found at follow-up 1 and 2, which coincided with the third wave of the SARS-CoV-2 pandemic in Portugal which led to the closure of schools again (follow-up 1) and the end of the school year (follow-up 2). Considering that teachers’ job demands and distress normally increase throughout the school year (e.g., von der Embse and Mankin, 2021), and that during this year these demands were exacerbated by the new waves of SARS-CoV-2, these results suggest that the *A+* may have contributed to a greater stability of SEC over time in the EG teachers and helped them to navigate across periods of greater uncertainty and adaptation.

Although the study hypotheses were not fully confirmed, the findings present promising preliminary evidence which may contribute to both research and practice in this field. Taken together, the findings of this study contribute to the knowledge on the potential of SEL interventions specifically designed for teachers. Particularly for cases where teachers perceive fewer personal resources and a more demanding working context, findings support that SEL interventions as the *A+* may offer an important contribution to the development of teachers’ personal resources to mitigate the negative impacts of job demands on their occupational health and well-being. Results also add to the current knowledge by emphasizing the importance of organizational climate on teachers’ SEC.

#### Findings on quality of the intervention implementation

As far as quality of intervention implementation is concerned and addressing our second research question (Q2), the findings indicate high levels of both facilitator and participants’ behaviors, across the three intervention groups. Moreover, no statistically significant differences were found with regards to the intervention implementation between the training groups. This is an important finding since, as previous literature depicts, the quality of the intervention implementation can affect and bias the intervention’s efficacy (Berkel et al., 2011, 2018; Humphrey et al., 2018). Then, particularly considering the mixed results found towards the *A+*’s efficacy, this is an important result since it allows to support the explanatory hypothesis that mixed results originate from between-context variability and not due to differences in the quality of the intervention.

Furthermore, our findings are in line with recent research that highlights the impact of participants’ responsiveness on the intervention’s outcomes (Berkel et al., 2018; Humphrey et al., 2018). When we look further into the participants’ responsiveness within each group, the results suggest that the active participation and engagement of the participants influenced the results within each EG. More specifically, Cluster A, where participants’ responsiveness did not seem to directly relate with their SEC development, was the context that showed the lowest group responsiveness (compared to Clusters B and C) and lowest within-group variability (*Interquartile range* = 2.17). Cluster B, on the other hand, showed the highest and most consistent group responsiveness (both active participation and attendance) throughout the intervention. And, along with Cluster C, presented a higher within-group variability (*Interquartile range* = 2.58), against Cluster A. This higher responsiveness appears to have made an important contribution to the perception of a greater development of SEC, particularly intrapersonal competence (i.e., self-awareness and self-regulation). Taken together, these results are in line with Berkel et al.’s (2011, 2018) model and contribute to the discussion on the importance of implementation quality research as a component of program planning with a view to enhancing and better understand programs’ efficacy.

### Limitations and future research

Despite its promising results, the present study also has limitations that warrant mention. A small, self-selected and geographically circumscribed sample was used which, adding to the role of the contextual variables, limits the generalization of the findings. However, to explore and comprehend the impact of the organizational climate on the teachers’ SEC was one of our main goals. And, as sustained by our results, the contextual variables predicted teachers’ SEC, and the school clusters that integrated this study presented significant differences between these contextual variables. Thus, we had to consider the different school clusters separately, notwithstanding the reduced sample size within each cluster. Nonetheless, within the three school-clusters assessed, after excluding teachers who did not comply with the eligibility criteria, and ensuring voluntary participation, we had a participants’ enrollment rate of 71.68%. The attrition rate is reasonable given how demanding the study was (i.e., investment in the 50 h of training, several moments of data collection, and participation during an entire school year). We have also chosen the data analysis procedures (i.e., use of robust statistics, account for family-wise error rate) seeking to minimize the impacts of this limitation. Taken together, in this study, to consider a small and geographically circumscribed sample was the best compromise to meet our goals. However, it calls for future studies to further validate the *A+* efficacy in different educational contexts, accounting for the necessary adaptations, and considering larger and more diverse samples. Still regarding our sample, the *A+* was developed for elementary-school teachers. However, given the scarcity of SEL interventions for teachers, it would be important for future studies to understand the suitability of *A+* to other groups of teachers (i.e., middle and high school, tertiary teachers). Also, with a larger sample, it would be interesting to test the mediation effects of the proximal variables on the distal variables to enable a better understanding of the relationship between the assessed outcomes, as well as the spillover effects to students’ SEC, well-being, and academic performance suggested by previous studies (Jennings and Greenberg, 2009; Carvalho et al., 2021).

On the other hand, only self-report measures were used to assess the intervention’s efficacy. Even though this was a means to ensure all data collection waves and reduce attrition due to the limitations caused by the SARS-CoV-2 pandemic, they are also more susceptible to SDB (Larson, 2019). A second issue with the use of self-report measures is the risk of a more conservative evaluation of one’s own competencies and symptoms, after gaining a more accurate perception of the variables under assessment. To account for this limitation, we have selected instruments previously studied with Portuguese samples, guaranteed anonymity and confidentiality of the responses, and included a statement encouraging honesty at the beginning of the data collection protocol (Larson, 2019). We have also included measures with different rating-scales and items with a reverse response direction to minimize the risk of acquiescent response bias (Rattray and Jones, 2007). Nevertheless, it is important for future research to use complementary data collection methods, such as behavioral and objective measures (e.g., situational judgment tests; Aldrup et al., 2020), to strengthen the evidence on the *A+* efficacy.

The use of an online intervention also bears constraints which could interfere with the program’s efficacy. Notwithstanding, having ensured learner-instructor interaction and connectedness, feedback opportunities and synchronous guidance, as well as user-friendly tools, which have been associated with high levels of user satisfaction and learning outcomes by prior research (Hofmann, 2014; Beatty and Binnion, 2016; Kintu et al., 2017), other aspects such as technological limitations and individual skills such as digital literacy may have still influenced the participants’ engagement in the program and outcomes (Hofmann, 2014; Kintu et al., 2017). Thus, when a period of greater stability is reached and the SARS-CoV-2 restrictions are lifted, thus allowing full access to the respective contexts, it would be important to test the *A+* efficacy in a face-to-face and/or blended learning format.

The fact that the participants were not blinded was also a limitation. The teachers may have raised their expectations of changes in the school by knowing that an intervention was occurring and, if those expected changes were not perceived to be met, worse evaluations can be made across time regarding the distal outcomes, namely in terms of well-being, stress, and burnout. Also, contamination across the treatment conditions cannot be overlooked since this study was performed in a real context in which the participants have relations with each other. Although this is a difficult constraint to overcome since, given the key role of context, it is important that teachers in the EG and CG belong to the same school cluster, future studies should try to account for this limitation. Lastly, regarding the treatment conditions, the use of a waitlist control group instead of an active control group can increase bias in the results. Voluntary participation could also lead to bias since teachers are already more available and aware of this issue. Thus, it is important for future research to resort to active comparison groups.

### Study impact

Despite the aforementioned limitations, this study advances important contributions to both research and practice. The findings provided promising indicators of the efficacy of the *A+* intervention program, a theoretically and empirically grounded online SEL intervention for teachers, particularly when considering the needs and characteristics of each intervention context. Results also explored the impact of quality of implementation. Taken together this study’s results contribute to filling the gap in the prior literature on SEL interventions for teachers (Oliveira et al., 2021a).

By presenting a between-subjects longitudinal design with four data collection waves across a school year and resorting to hierarchical models to test interaction effects, this study contributes to more methodologically robust research regarding SEL interventions for teachers (Oliveira et al., 2021a). By sustaining the predictive value of organizational climate dimensions on teachers’ SEC, our results also corroborate previous studies which stress the prominent impact of contextual variables on teachers’ personal and professional outcomes (namely, SEC development and occupational health; Schaufeli and Taris, 2014; Collie, 2017). In our study, most of the organizational climate dimensions assessed predicted at least one dimension of teachers’ SEC (both at an intrapersonal and interpersonal level) at baseline. Thus, our findings reinforce the interaction between personal-level and organizational-level demands and resources (Granziera et al., 2021). Consequently, they also highlight the need to consider teachers’ occupational health and well-being as being multidimensionally impacted (i.e., intra-, inter-personally and organizationally) when designing and assessing policies and interventions to mitigate teachers’ ill-health (Schaufeli and Taris, 2014; Collie, 2017; Taris et al., 2017; Ford et al., 2019). Taken together, these results support and highlight the importance of adopting a systemic approach in interventions aimed at promoting teachers’ occupational health. As the findings strengthen the important role of schools’ environment and educational systems for teachers’ occupational health and well-being, then it is understandable that teachers cannot be solely held accountable (Granziera et al., 2021). Similarly to what literature has highlighted for other professions with a high prevalence of burnout (e.g., physicians; Jha et al., 2018), it is essential to look at teachers’ occupational health and burnout as a socio-professional problem that requires intervention directed, complementarily, at both individual and contextual/organizational level dimensions. Consequently, there is need for SEL interventions to adopt a whole-school approach involving all the school personnel (Durlak et al., 2015; Collie, 2020).

Moreover, by using independent observational data grounded in Berkel et al.’s (2011, 2018) model, this study also contributes to further exploring the relationship between SEL interventions’ quality of implementation and efficacy. When assessing quality of implementation, prior studies have mostly focused on the impact of fidelity on program outcomes (Oliveira et al., 2021a). However, recent research has brought a new perspective to the table where, above facilitator behaviors (the main impact of which appears to be on participants’ responsiveness), program outcomes are directly impacted by participants’ responsiveness (Berkel et al., 2018; Humphrey et al., 2018). Although preliminary, our findings (in which the participants’ active participation and engagement seems to have influenced the outcomes on EG teachers’ SEC development) align with this study and sustain the importance of participants’ responsiveness.

Additionally, this study makes available a theoretically grounded, valid, and culturally adapted intervention, which is specific to teachers’ needs and allows teachers to see direct personal and professional benefits (Granziera et al., 2021). By effectively promoting teachers’ personal resources (e.g., self-regulation, positive relations and conflict management skills) to face job demands, the *A+* can be a useful resource to help break the “vicious circle” of teachers’ burnout symptoms (e.g., increasing well-being and decreasing emotional exhaustion symptoms; Guthier et al., 2020; Bianchi et al., 2021). Moreover, by presenting good indicators of efficacy across time (and in a particularly demanding period), this intervention can also contribute to reducing costs (temporal and financial) in educational contexts by being able to provide teachers with effective resources to manage personal and professional challenges over time. Furthermore, considering the instability experienced during the SARS-CoV-2 pandemic, online interventions such as the *A+* may become a flexible and refined tool to promote teachers’ occupational health and well-being. This online format enables the adaptation and continuity of the interventions even in situations where the participants are isolated or in new lockdowns, thus continuing to support teachers in moments of transition/greater demand and instability. As previously mentioned, there may have been a ceiling effect impacting intervention’s efficacy at Cluster B. If true, this result signals the importance to do not develop only universal interventions, as it was the case of the *A+* intervention. Thus, it is important that researchers and practitioners develop and assess the impacts of multi-tiered interventions (Oliveira et al., 2021a), for example that follows a targeted universalism approach in which differentiated adaptations are needed to attain the desired outcomes (e.g., Powell et al., 2019). Lastly, the longitudinal analysis revealed sleeper effects (e.g., for both Cluster A and C, emotional regulation skills of the EG only increase significantly from follow-up 1) and fragile stability (with fluctuations in the outcome variables, e.g., in Cluster C, teachers from the EG describe a significant decrease in their occupational stress at follow-up 1 that regresses at follow-up 2) of the *A+* impacts across the data collection waves, thus reinforcing the importance of developing SEL interventions which are embodied in the school-contexts to allow for regular monitorization, skillful feedback and booster sessions.

## Data availability statement

 The datasets generated for this study and the codebooks which underlie the analyses can be found in the Open Science Framework repository: https://doi.org/10.17605/OSF.IO/2KZGW.

## Ethics statement

The studies involving human participants were reviewed and approved by Scientific and Ethical Council of the Faculty of Psychology, University of Lisbon. The patients/participants provided their written informed consent to participate in this study.

## Author contributions

SO designed and executed the study, analyzed the data, and wrote, edited, and revised the manuscript. MR assisted with the design of the study and the data analyses, and collaborated with the writing and the editing of the final manuscript. AV-S assisted with the design and execution of the study, and the editing of the final manuscript. AM-P assisted with the design, execution, and theoretical grounding of the study, and collaborated with the writing and the editing of the final manuscript. All authors contributed to the article and approved the submitted version.

## Funding

This work received national funding from FCT - Fundação para a Ciência e a Tecnologia, I.P., through a PhD grant (SFRH/BD/137845/2018) and through the Research Center for Psychological Science of the Faculty of Psychology, University of Lisbon (CICPSI; UIDB/04527/2020 and UIDP/04527/2020).

## Conflict of interest

The authors declare that the research was conducted in the absence of any commercial or financial relationships that could be construed as a potential conflict of interest.

## Publisher’s note

All claims expressed in this article are solely those of the authors and do not necessarily represent those of their affiliated organizations, or those of the publisher, the editors and the reviewers. Any product that may be evaluated in this article, or claim that may be made by its manufacturer, is not guaranteed or endorsed by the publisher.

## Footnotes

^1^ https://www.qualtrics.com
