# Supplementary material for: Effects of the A+ intervention on elementary-school teachers’ social and emotional competence and occupational health
Source: Front Psychol. 2022 Oct 12;13:957249. doi: 10.3389/fpsyg.2022.957249 (PMC9596939; doi:10.3389/fpsyg.2022.957249)
Supplement: Supplementary file 1 [file Data_Sheet_1.pdf]

Supplementary Online Material

Expanded methodological procedures and Complementary results

[To accompany Oliveira, S., Roberto, M. S., Veiga-Simão, A. M., & Marques-Pinto, A. (2022). Effects of the A+ intervention on elementary-school teachers' social and emotional competence and occupational health. *Frontiers in Psychology*, *13*, Article 957249. https://doi.org/10.3389/fpsyg.2022.957249]

The following supplementary materials include: greater detail on the contents and structure of the intervention program being studied, and complementary results regarding univariate and bivariate descriptive statistics within school clusters.

1 Expanded Methodological Procedures

1.1 Intervention full description

The *A+* is an online intervention program which sought to promote teachers' social and emotional competence. It builds on a prior study which tackles the planning of the intervention by means of a needs assessment within the intervention contexts and a pilot study to evaluate the social validity and efficacy of a trial version of the program (Oliveira et al., under revision). The SEL framework for teachers (Durlak et al., 2015; Elias et al., 1997; Jennings and Greenberg, 2009), the JD-R model (Demerouti et al., 2001; Schaufeli and Taris, 2014), and Collie's (2020) Social and Emotional Competence School model ensured theoretical ground for the development of the *A+*'s contents and methodologies. While findings from previous studies on SEL interventions' efficacy (Carvalho et al., 2021; Collie et al., 2012, 2018; Jennings et al., 2013, 2017; 2019; Oliveira et al., under revision) and on good practices for effective SEL (Durlak et al., 2010) and online (Beatty & Binnion, 2016; Hofmann, 2014; Kintu et al., 2017) interventions ensured the empirical ground for it.

To be more precise, the SEL framework for teachers (Durlak et al., 2015; Elias et al., 1997; Jennings and Greenberg, 2009) informed on specific SEC domains that should be addressed (i.e., self-awareness, self-regulation, social awareness, relationship skills and responsible decision-making), and on specific behavioral and motivational strategies to promote the desired behavioral change (e.g., problem-focused coping strategies). The JD-R model (Demerouti et al., 2001; Schaufeli and Taris, 2014) provided support for the teacher-specific stressors and their associations with the variables that are expected to be impacted by this intervention (e.g., teacher burnout). Lastly, Collie's (2020) Social and Emotional Competence School model depict an interactive process in which the development of SEC stem from an urge for autonomy, competence and relatedness need satisfaction, and that is influenced by the individual's context and perceived social support. Hence, training groups were built within the same school cluster and different group activities were proposed to increase relatedness and teachers' social support networks among peers (Oliveira et al., under revision).

Regarding its contents, and following the previously described theoretical and empirical evidence, the *A+* aimed to promote teachers' self-awareness, self-regulation, social awareness,

relationship skills and responsible decision-making. To this end, the intervention program includes five components, organized on the basis of stress-generating situations (Oliveira et al., under revision): *Personal organization and time management*, *Emotional awareness and regulation, Conscious communication, Conflict management*, and *Personal leadership*.

The *personal organization and time management* module involved enhancing teachers' competences to be able to balance and respond to both work and personal life commitments, through the development of self-awareness and self-regulation skills. Within this component, teachers developed practical strategies to help them set and achieve their goals and trained their adaptability skills in order to feel more comfortable to welcome change and adapt in the face of new information or situations. They also had the opportunity to learn different approaches to organizing their time, work and personal space to respond to different daily demands/tasks maintaining focus/energy (through emotional, behavioral and motivational regulation). Through these activities, teachers may become more efficient in defining their priorities during the day and optimize their productivity, which contributes to a higher work-life balance.

The *emotional awareness and regulation* component comprehended experiential and reflective exercises which addressed themes such as understanding and recognizing one's emotions, feelings, and emotional expressions (physiological, cognitive, and behavioral) towards other people and everyday situations, as well as the self-regulation of one's emotions. These activities may help teachers to develop more accurate self-perception and contribute to their self-efficacy, optimism, and resilience. This module sought to promote teachers' awareness of their individual characteristics, emotions and behaviors, and their ability to self-regulate their own emotions and consequent behaviors and decisions in both regular and challenging situations.

Within the *conscious communication* sessions, the themes focused on the ability to build and maintain strong and supportive relationships and interact effectively with others. Through the proposed exercises, the participants were challenged to identify different communication patterns (in different situations and contexts, but also observing different people), to take perspective and recognize different behavior functions, and consequently to train their assertiveness, active listening, and empathy. The exercises may stimulate teachers' attention, awareness, and ability to recognize others' emotions through the observation of their (non-)verbal communication, but also incite teachers' ability to say no and authentically express their own perspectives, needs, opinions, and feelings. The aim of these training sessions was to promote teachers' open communication which contributes to their ability to build positive relationships and to collaborate with others.

On the other hand, the *conflict management* component may foster the development of skills which enable teachers to effectively prevent and manage conflict situations and negative social interactions. Following on from the initial exercises addressed in the previous module, teachers were asked to not only recognize different perspectives but also to go beyond and comprehend, appreciate, respect and value those diverse perspectives and opinions even when facing conflicting situations. The importance of focusing on the specific behavior and not on generalizations was also approached, as well as the ability to openly admit personal mistakes/misbehaviors, to be receptive to others' criticism/feedback, and to act and communicate without resentment. Towards the end of this module, group exercises were

performed to actively train teachers' collaborative strategies, and to help teachers work in groups to find common solutions while respecting others.

The *personal leadership* module practices were intended to promote teachers' ability to make ethical and constructive decisions, evaluate and reflect on their behaviors, and to effectively solve problems. This is an integrative component of the training course where themes such as the importance of developing a growth mindset towards behavior evaluation and self-reflection, the ability to correctly identify a problem and to generate multiple solutions to effectively solve everyday problems, and the need to integrate both intrapersonal and interpersonal skills to make decisions that are personal, moral, and ethically responsible, were covered.

Overall, the *A+* was designed to consider teachers as a whole, with different but interdependent life roles. Hence, all the sessions of the intervention program were tailored to cover teachers' personal, social, and professional aspects of life. Throughout the training course, the teachers were invited to follow a ladder-like path. In the first two modules, emphasizing intrapersonal skills, the participants worked to gain awareness and become more able to regulate their emotions and behaviors in an autoscopic process. Only then, in a second step, did the contents address interpersonal skills. In the third and fourth components, the teachers began to work on increasing their awareness of how they feel and behave when they interact with others and to develop strategies to communicate more intentionally and effectively. Lastly, in the final step, *Personal leadership* emerges as an integrative module where all the previously competences are intertwined to help teachers create optimal experiences, aligned with their goals and needs while also respecting others, and balancing their different life roles. The name *A+* was also thought to highlight the importance of a balanced life. At a first glance, *A+* suggests that participants are in a process of becoming a teacher of excellence (worthy of the top grade). However, in Portuguese, the meaning of *A+* when read aloud is "there is more than…", which throughout the intervention worked as a reminder that teachers are more than their professional role, and their life experiences outside work also matter and influence their performance.

Regarding its structure, the *A+* consisted of a total of 50 hours of training, 25 of which were delivered in 10 weekly 2.5-hour in-group synchronous sessions and 25 of which consisted in asynchronous training. Each component of the A+ had approximately five hours of synchronous training (i.e., two training sessions). Moreover, the intervention program followed the SAFE guidelines for interventions (i.e., sequenced training activities; active learning methods; focus on the development of SEC; and explicit SEL aims; Durlak et al., 2010). Thus, all the sessions included both the following expositive and active moments: introduction of new concepts, individual and group reflections, role-playing, storytelling and readings, metaphors/analogies, feedback moments, brainstorming, written exercises, lectures, group chats, testimonies, video viewing and homework assignments (the so-called "weekly challenges"). The 25 hours of synchronous training sessions were delivered through the *Zoom* software. The 25 hours consisting of asynchronous training were supported by the *Moodle* platform where participants could find support for the weekly challenges (one exercise per week, related to the contents covered in the synchronous session and which aimed to help teachers implement the competences and skills that had been addressed in their daily lives), complementary reading resources and a group chat where they could share reflections, doubts,

insights and other complementary materials with the other participants and the facilitator. Figures S1, S2 and S3 illustrate, respectively, an example of an activity presented in a synchronous session at *Zoom*; a weekly challenge to be performed during the asynchronous training hours; and a screenshot of the *Moodle* environment where teachers could find all support materials and the group chat.

Lastly, since the intervention program took place exclusively online, literature recommendations on the best practices to ensure quality and efficacy of online interventions also informed the procedures of the *A+* (Beatty & Binnion, 2016; Hofmann, 2014; Kintu et al., 2017). Specifically: (1) learner-instructor interaction and connectedness throughout the intervention, either at the synchronous as the asynchronous training hours, was ensured; (2) synchronous guidance and feedback opportunities were guaranteed for both the contents and activities developed within the synchronous sessions, and the weekly challenges and complementary resources that were made available in the *Moodle* platform; (3) parsimonious and user-friendly tools were selected. Furthermore, throughout the synchronous sessions, regular moments for interaction (either an active moment or a moment for questions) were ensured to retain participants' attention and engagement. Participants' progress was also weekly monitored to ascertain that the intervention's contents were being followed. Moreover, at the beginning of the 1st synchronous session, the platforms and its specific resources were presented to the participants (e.g., how to access and use the group chat), as well as the completion requirements. Besides these main resources, the remaining functionalities of the platforms (e.g., parallel rooms for small working groups, submission of documents for final evaluation) were gradually introduced throughout the intervention. Additionally, a visual course map with the intervention schedule was made available for the participants from session 1, and the *Moodle* platform was also organized following this map. Participants were also provided with a direct phone number, through which they could ask for support to work through logistical and/or technical difficulties.

Figure S1
The activity “*Wheel of Feelings*” presented in a synchronous session at *Zoom*.

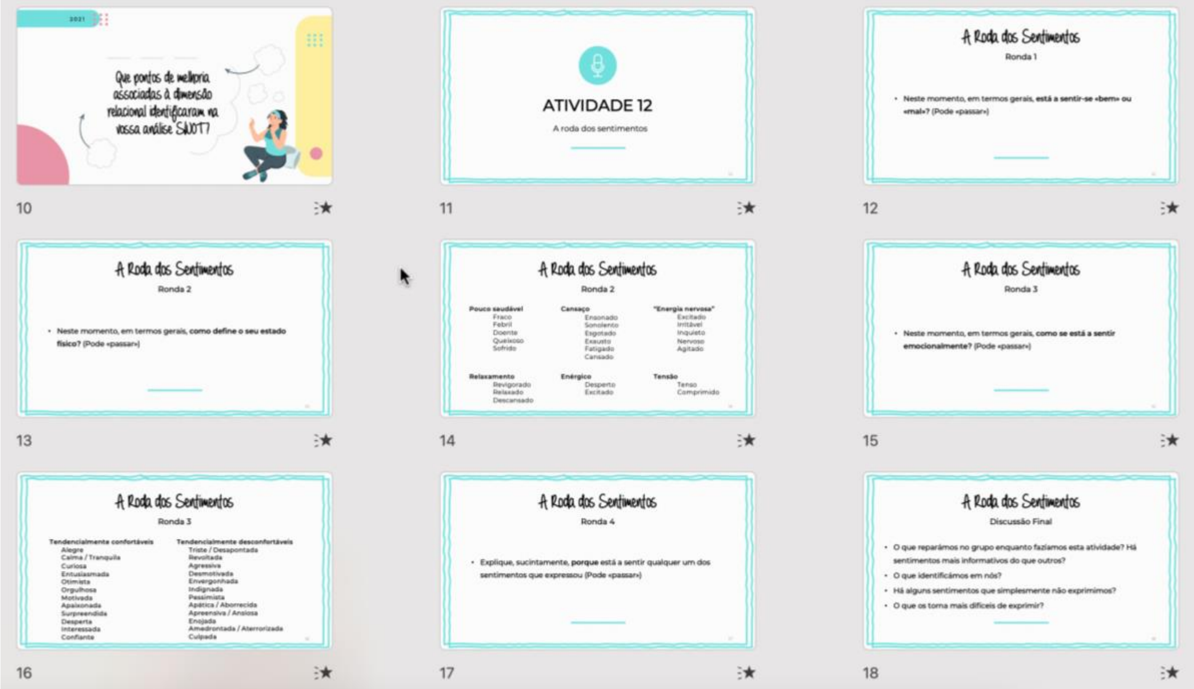

Figure S2
Segment of the activity sheet which supports the weekly challenge "*CEO of my life*".

Relembre os diferentes departamentos da "empresa" que é:

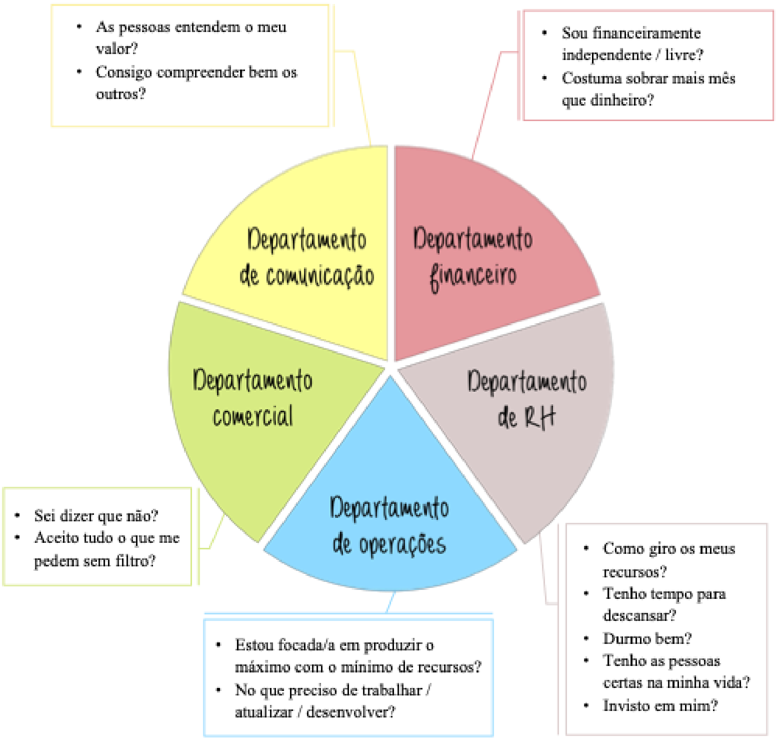

Sendo 10 a pontuação máxima e 0 a mínima, qual a avaliação que atribui (neste momento) a cada departamento da sua empresa?

Enquanto Diretor/a Executivo/a o que pode fazer para melhorar este departamento?

0-10 Departamento Financeiro: _____

O que falta para ser 10?

O que posso fazer, agora, para melhorar este departamento?

Figure S3
Screenshot of different segments of the *Moodle* environment

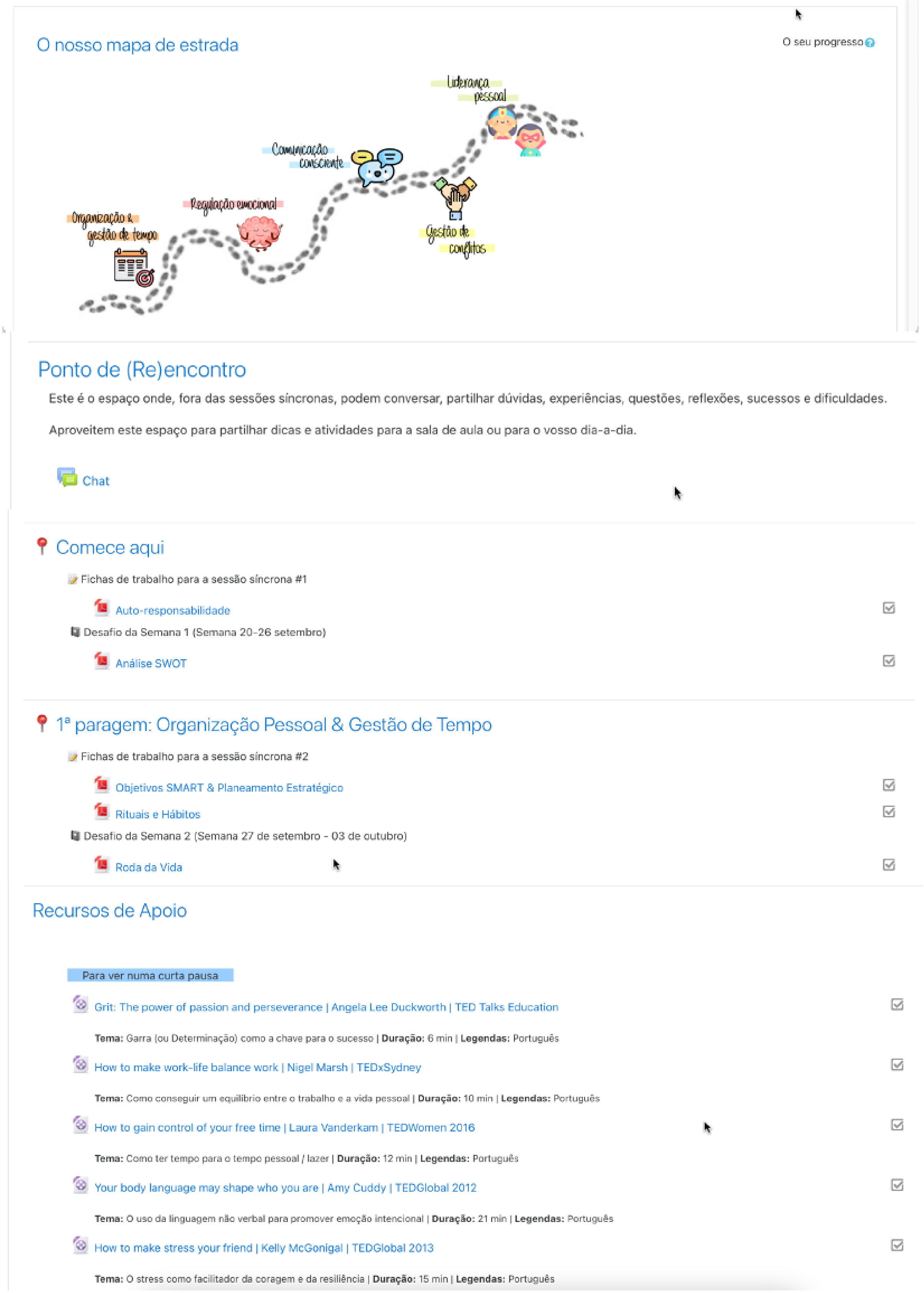

2. Expanded Results Regarding Univariate and Bivariate Descriptive Statistics Within School Clusters

Table S1

Spearman Correlation Coefficients Between Organizational Climate Dimensions and Proximal Dependent Variables Assessed (Affect, Emotion Regulation, and Social and Emotional Competence) (N = 81)

	Positive affect T1	Negative affect T1	Cognitive reappraisal T1	Expressive suppression T1	Self-regulation T1	Self-awareness T1	Conflict management T1	Positive relationship T1	Responsible decision making T1
Professional relationships management T1	.03	-.15	-.11	.11	.05	.02	.14	.11	.16
Pedagogical tasks management T1	.08	.14	.05	.25*	-.02	-.12	-.23	-.03	-.05
Bureaucratic tasks management T1	.22*	-.23*	-.05	.07	-.07	.03	-.02	-.04	.04
Professional interactions among teachers T1	.18	-.16	.08	-.03	-.26*	.09	.26*	.37**	.18
Personal interactions among teachers T1	.21*	-.03	.12	-.01	.04	-.07	-.18	.12	.00
Dynamic of the teacher’s group T1	-.05	-.23*	-.24*	-.17	-.01	-.04	.40**	.23*	.10

* $p < .05$, ** $p < .01$

Table S2

Means and Standard Deviations of Teacher Self-Report of Affect, Emotion Regulation, Social and Emotional Competence, Self-care, Sleep Quality, Well-being, and Burnout at the Four Data Collection Waves – Cluster A (n = 19)

	Experimental Group				Control Group			
Outcome variable	Pretest	Posttest	Follow-up 1	Follow-up 2	Pretest	Posttest	Follow-up 1	Follow-up 2
**Affect**	*M(SD)*	*M(SD)*	*M(SD)*	*M(SD)*	*M(SD)*	*M(SD)*	*M(SD)*	*M(SD)*
Positive affect	3.37 (0.67)	3.29 (0.67)	3.47 (0.58)	3.03 (0.79)	3.21 (0.64)	2.95 (0.79)	3.29 (0.57)	2.78 (0.52)
Negative affect	2.08 (0.71)	1.76 (0.67)	1.62 (0.67)	1.73 (0.57)	1.83(0.61)	1.80(0.59)	1.76(0.80)	1.91(1.02)
**Emotion regulation**								
Cognitive reappraisal	4.64 (1.10)	4.82 (0.89)	4.76 (0.88)	5.20 (0.90)	5.21(0.73)	5.13(1.09)	4.89(0.75)	4.54(1.04)
Expressive suppression	3.73 (1.21)	3.33 (1.02)	3.40 (0.89)	3.14 (1.58)	3.31(0.73)	3.46(0.63)	3.75(1.00)	3.43(1.06)
**Social and emotional competence**								
Self-regulation	3.46 (0.47)	3.57 (0.46)	3.65 (0.32)	3.43 (0.39)	3.39(0.54)	3.30(0.33)	3.30(0.35)	3.22(0.30)
Self-awareness	3.90 (0.31)	3.73 (0.45)	3.79 (0.38)	3.75 (0.41)	4.13 (0.32)	3.95 (0.38)	3.71 (0.40)	3.55 (0.34)
Positive relationship	3.68 (0.28)	3.55 (0.25)	3.44 (0.29)	3.52 (0.28)	3.77 (0.46)	3.66 (0.46)	3.63 (0.53)	3.66 (0.36)
Conflict management	3.52 (0.33)	3.58 (0.42)	3.45 (0.35)	3.50 (0.34)	3.64 (0.48)	3.66 (0.36)	3.55 (0.29)	3.61 (0.31)
Responsible decision making	3.59 (0.37)	3.65 (0.39)	3.54 (0.36)	3.48 (0.47)	3.67 (0.45)	3.56 (0.43)	3.63 (0.34)	3.56 (0.31)
**Self-care**	2.94 (0.88)	2.71 (1.13)	2.81 (0.70)	2.66 (0.85)	2.94 (0.70)	2.19 (0.78)	2.38 (0.78)	2.84 (1.05)
**Sleep quality**	2.75 (0.75)	3.08 (0.90)	3.00 (0.60)	2.91 (0.83)	2.75 (1.04)	2.75 (0.71)	3.00 (0.76)	2.50 (0.54)
**Well-being**								
Emotional well-being	3.67 (0.77)	3.78 (0.77)	4.02 (0.74)	3.48 (0.99)	3.37 (0.81)	3.21 (1.05)	3.25 (0.71)	3.37 (1.24)

Psychological well-being	3.12 (1.19)	3.33 (0.92)	3.65 (0.74)	3.09 (1.05)	3.06 (1.07)	2.60 (1.04)	2.96 (0.82)	2.92 (0.93)
Social well-being	2.40 (1.01)	2.40 (0.83)	2.98 (0.85)	2.62 (1.10)	3.23 (0.95)	1.80 (0.99)	2.48 (0.93)	2.13 (0.92)
**Occupational stress**	3.83 (0.72)	3.67 (0.89)	3.42 (1.24)	3.91 (0.83)	4.50 (0.76)	4.38 (1.06)	4.00 (0.93)	4.50 (0.76)
**Burnout symptoms**								
Emotional exhaustion	2.93(1.08)	2.54(1.01)	2.37(1.22)	2.82(1.21)	3.04(1.05)	3.54(1.49)	3.00(1.35)	3.42(1.53)
Depersonalization	0.47 (0.62)	0.55 (0.60)	0.52 (0.78)	0.84 (0.94)	0.58 (0.67)	1.00 (0.91)	0.85 (1.70)	1.18 (1.13)
Personal accomplishment	4.51 (0.57)	4.42 (0.78)	4.54 (0.82)	4.17 (0.74)	4.36 (0.57)	4.52 (0.54)	4.43 (0.77)	4.47 (0.77)

Table S3

Means and Standard Deviations of Teacher Self-Report of Affect, Emotion Regulation, Social and Emotional Competence, Self-care, Sleep Quality, Well-being, and Burnout at the Four Data Collection Waves – Cluster B (n = 19)

Outcome variable	Experimental Group				Control Group			
	Pretest	Posttest	Follow-up 1	Follow-up 2	Pretest	Posttest	Follow-up 1	Follow-up 2
**Affect**	*M(SD)*	*M(SD)*	*M(SD)*	*M(SD)*	*M(SD)*	*M(SD)*	*M(SD)*	*M(SD)*
Positive affect	3.50 (0.78)	3.17 (0.82)	3.30 (0.80)	3.30 (0.80)	3.44 (0.42)	3.46 (0.46)	3.03 (0.61)	2.83 (0.98)
Negative affect	1.60 (0.65)	1.64 (0.57)	1.31 (0.36)	1.34 (0.30)	1.71 (0.43)	1.36 (0.24)	1.58 (0.50)	1.42 (0.37)
**Emotion regulation**								
Cognitive reappraisal	5.00 (1.11)	5.28 (0.87)	5.12 (1.16)	5.07 (1.41)	4.87 (1.02)	5.09 (1.29)	4.56 (0.95)	4.46 (1.21)
Expressive suppression	3.33 (1.26)	3.44 (1.19)	3.45 (1.49)	3.28 (1.11)	2.94 (0.61)	3.33 (1.29)	3.67 (0.75)	3.19 (1.15)
**Social and emotional competence**								
Self-regulation	3.59 (0.53)	3.64 (0.54)	3.60 (0.62)	3.51 (0.60)	3.43 (0.24)	3.49 (0.51)	3.33 (0.55)	3.32 (0.48)
Self-awareness	4.02 (0.47)	3.92 (0.53)	3.92 (0.54)	3.86 (0.42)	3.89 (0.31)	3.86 (0.27)	3.84 (0.30)	3.87 (0.42)
Positive relationship	3.89 (0.36)	3.92 (0.45)	3.88 (0.45)	3.93 (0.41)	3.65 (0.46)	3.71 (0.39)	3.85 (0.27)	3.65 (0.29)
Conflict management	3.79 (0.44)	3.79 (0.46)	3.68 (0.55)	3.71 (0.53)	3.58 (0.42)	3.65 (0.53)	3.50 (0.46)	3.56 (0.46)
Responsible decision making	4.04 (0.45)	4.03 (0.34)	3.92 (0.43)	3.95 (0.41)	3.76 (0.49)	3.78 (0.37)	3.59 (0.25)	3.50 (0.34)
**Self-care**	2.96 (0.81)	2.67 (1.04)	2.52 (0.75)	2.58 (1.06)	3.06 (0.66)	2.67 (0.35)	2.75 (0.60)	2.83 (0.54)
**Sleep quality**	2.62 (0.77)	2.75 (0.45)	2.73 (0.47)	2.70 (0.48)	2.78 (0.44)	2.67 (0.71)	2.89 (0.33)	2.67 (0.71)
**Well-being**								
Emotional well-being	3.23 (1.20)	3.19 (1.14)	3.21 (1.13)	2.63 (1.25)	3.89 (0.69)	3.67 (0.71)	3.70 (0.45)	3.11 (0.82)

Psychological well-being	3.22 (0.99)	3.13 (1.21)	3.11 (1.34)	2.97 (1.42)	3.26 (0.83)	3.15 (0.57)	2.94 (0.79)	3.20 (0.82)
Social well-being	2.48 (0.94)	2.08 (0.97)	2.80 (2.07)	2.10 (1.14)	2.49 (0.87)	2.42(0.86)	2.44 (0.90)	2.33 (1.11)
**Occupational stress**	3.54 (1.27)	3.83 (1.27)	3.82 (0.98)	4.10 (1.10)	3.67 (1.12)	4.00 (0.87)	4.22 (0.67)	4.22 (0.67)
**Burnout symptoms**								
Emotional exhaustion	2.72 (1.32)	2.81 (1.13)	2.82 (1.14)	3.22 (1.04)	2.84 (0.57)	2.90 (0.95)	3.11 (0.70)	3.19 (0.91)
Depersonalization	0.49 (0.71)	0.37 (0.59)	0.46 (0.72)	0.22 (0.24)	0.78 (0.76)	0.82 (0.76)	0.51 (0.62)	0.67 (0.73)
Personal accomplishment	4.89 (0.75)	4.72 (0.49)	4.57 (0.92)	4.72 (0.81)	4.61 (0.44)	4.69 (0.71)	4.69 (0.21)	4.25 (0.69)

Table S4

Means and Standard Deviations of Teacher Self-Report of Affect, Emotion Regulation, Social and Emotional Competence, Self-care, Sleep Quality, Well-being and Burnout at the Four Data Collection Waves – Cluster C (n = 36
)

	Experimental Group				Control Group			
Outcome variable	Pretest	Posttest	Follow-up 1	Follow-up 2	Pretest	Posttest	Follow-up 1	Follow-up 2
**Affect**	*M(SD)*	*M(SD)*	*M(SD)*	*M(SD)*	*M(SD)*	*M(SD)*	*M(SD)*	*M(SD)*
Positive affect	3.02 (0.66)	3.31 (0.38)	3.02 (0.57)	3.02 (0.57)	3.37 (0.44)	3.28 (0.62)	3.36 (0.73)	3.07 (0.73)
Negative affect	1.82 (0.77)	1.70 (0.55)	1.65 (0.59)	1.75 (0.74)	1.77 (0.83)	1.71 (0.73)	1.67 (0.75)	1.61 (0.49)
**Emotion regulation**								
Cognitive reappraisal	4.92 (0.96)	5.08 (0.94)	5.06 (0.81)	4.99 (1.07)	4.16 (1.33)	4.84 (1.11)	4.18 (1.72)	4.96 (1.26)
Expressive suppression	4.00 (1.37)	3.87 (1.22)	4.04 (1.10)	3.72 (1.25)	2.58 (1.11)	3.17 (1.45)	3.03 (1.46)	3.21 (1.45)
**Social and emotional competence**								
Self-regulation	3.58(0.49)	3.37 (0.39)	3.47 (0.49)	3.50 (0.53)	3.70 (0.44)	3.67 (0.41)	3.68 (0.45)	3.68 (0.56)
Self-awareness	3.97 (0.42)	3.82 (0.29)	3.89 (0.42)	3.77 (0.51)	3.99 (0.50)	3.89 (0.44)	3.93 (0.48)	3.95 (0.51)
Positive relationship	3.60 (0.43)	3.71 (0.51)	3.79 (0.61)	3.74 (0.50)	3.89 (0.48)	3.76 (0.48)	3.80 (0.43)	3.81 (0.43)
Conflict management	3.60 (0.43)	3.57 (0.50)	3.68 (0.41)	3.75 (0.53)	3.84 (0.42)	3.63 (0.48)	3.62 (0.38)	3.64 (0.45)
Responsible decision making	3.75 (0.38)	3.75 (0.44)	3.75 (0.49)	3.71 (0.54)	3.80 (0.44)	3.75 (0.46)	3.73 (0.39)	3.75 (0.43)
**Self-care**	2.41 (0.78)	2.43 (0.51)	2.60 (1.06)	3.10 (1.04)	2.99 (0.91)	2.67 (0.92)	2.45 (0.84)	2.57 (0.96)
**Sleep quality**	2.59 (0.87)	2.82 (0.81)	2.88 (0.60)	2.88 (0.60)	2.95 (0.72)	3.10 (0.63)	2.84 (0.60)	2.89 (0.57)
**Well-being**								
Emotional well-being	3.53 (1.03)	4.07 (0.57)	3.84 (0.68)	3.84 (0.83)	4.24 (0,22)	3.88 (0.69)	4.01 (0.79)	3.79 (0.71)

Psychological well-being	3.42 (1.02)	3.59 (0.87)	3.31 (1.21)	3.46 (0.94)	3.52 (0.86)	3.47 (0.83)	3.43 (0.91)	3.52 (0.77)
Social well-being	2.36 (1.21)	2.80 (0.93)	2.76 (0.97)	3.11 (0.78)	2.61 (0.89)	2.54 (0.85)	2.58 (0.85)	2.74 (0.65)
**Occupational stress**	4.12 (0.99)	3.88 (0.99)	3.41 (0.87)	3.76 (0.97)	3.86 (0.94)	3.71 (0.96)	3.84 (0.90)	3.41 (1.22)
**Burnout symptoms**								
Emotional exhaustion	3.10 (1.34)	3.11 (1.24)	2.88 (1.34)	2.89 (1.24)	2.91 (1.59)	2.40 (1.29)	2.26 (1.31)	2.54 (1.26)
Depersonalization	0.76 (1.17)	0.91 (1.15)	0.77 (1.22)	0.88 (1.48)	0.63 (1.23)	0.46 (0.79)	0.36 (0.69)	0.36 (0.62)
Personal accomplishment	4.64 (0.84)	4.74 (0.65)	4.64 (0.80)	4.78 (0.57)	4.95 (0.65)	4.88 (0.75)	4.76 (0.96)	4.87 (0.94)
